# Genetic and Ecological Diversity of *Escherichia coli* and Cryptic *Escherichia* Clades in Subtropical Aquatic Environments

**DOI:** 10.3389/fmicb.2022.811755

**Published:** 2022-02-17

**Authors:** Xiu Pei Koh, Zhiyong Shen, Chun Fai Woo, Yanping Yu, Hau In Lun, Sze Wan Cheung, Joseph Kai Cho Kwan, Stanley Chun Kwan Lau

**Affiliations:** ^1^Division of Environment and Sustainability, The Hong Kong University of Science and Technology, Kowloon, Hong Kong SAR, China; ^2^Department of Ocean Science, The Hong Kong University of Science and Technology, Kowloon, Hong Kong SAR, China; ^3^Division of Life Science, The Hong Kong University of Science and Technology, Hong Kong, Hong Kong SAR, China

**Keywords:** *Escherichia*, genetic diversity, subtropical, aquatic environment, cryptic clades, environmental *E. coli*, ecological differentiation, human impact

## Abstract

*Escherichia coli* not only inhabit the large intestines of human and warm-blooded animals but could also persist in the external environment. However, current knowledge was largely based on host-associated strains. Moreover, cryptic *Escherichia* clades that were often misidentified as *E. coli* by conventional diagnostic methods were discovered. Failure to distinguish them from *E. coli sensu stricto* could lead to inaccurate conclusions about the population genetics of *E. coli*. Based on seven housekeeping genes, we determine the genetic and ecological diversity of *E. coli* and cryptic clades as they occupy aquatic habitats with different characteristics and human impact levels in subtropical Hong Kong. Contrary to previous reports, clade II was the most abundant cryptic lineage co-isolated with *E. coli*, being especially abundant in relatively pristine subtropical aquatic environments. The phylogenetically distinct cryptic clades and *E. coli* showed limited recombination and significant genetic divergence. Analyses indicated that these clade II strains were ecologically differentiated from typical *E. coli*; some may even represent novel environmental *Escherichia* clades that were closely related to the original clade II strains of fecal origins. *E. coli* of diverse origins exhibited clonality amidst divergent genotypes STs, echoing other studies in that recombination in housekeeping genes was insufficient to disrupt phylogenetic signals of the largely clonal *E. coli*. Notably, environmental *E. coli* were less diverse than fecal isolates despite contributing many new alleles and STs. Finally, we demonstrated that human activities influenced the distribution of *E. coli* and clade II in a small aquatic continuum. Moving from relatively pristine sites toward areas with higher human disturbance, the abundance of clade II isolates and new *E. coli* genotypes reduces, while *E. coli* bearing class I integrons and belonging to CCs of public health concern accumulates. Altogether, this work revealed the new genetic diversity of *E. coli* and cryptic clades embedded in selected subtropical aquatic habitats, especially relatively pristine sites, which will aid a more thorough understanding of the extent of their genetic and functional variations in relation to diverse habitats with varied conditions.

## Introduction

Variations in DNA sequences underlie the evolution and divergence of the physiological, metabolic, and functional traits of organisms as they adapt to different habitats (Maiden et al., 1998; Spratt, 1999; Reid et al., 2000; Schloter et al., 2000; Yang et al., 2004; Tenaillon et al., 2010). Thus, probing the correspondence between genetic diversification and ecological differentiation is fundamental to construe the evolutions that caused the formation of permanently separated genetic clusters of closely related bacteria with respective ecological roles (Fraser et al., 2007; Hunt et al., 2008). Ecological differentiation occurs not only between species but also within species (Cohan and Perry, 2007; Hunt et al., 2008; Coleman and Chisholm, 2010). Many bacterial species contained multiple ecologically distinct populations, as observed in *Escherichia coli* (Gordon and Cowling, 2003; Touchon et al., 2020), *Legionella pneumophila* (Cianciotto, 2006), *Vibrio splendidus* (Hunt et al., 2008), *Bacillus simplex* (Koeppel et al., 2008), etc.

Current knowledge about the genus *Escherichia* largely stems from its type species, *E. coli*, which commonly inhabits the gastrointestinal tracts of human and warm-blooded animals (primary habitats). While many strains are considered transient in external environments (secondary habitats) after being egested from their hosts, some could persist or even become naturalized in diverse environmental matrices such as waters, soils, sediments, and periphyton (Ishii and Sadowsky, 2008; Touchon et al., 2020). In line with its diverse habitats, *E. coli* displays considerable phenotypic and genetic variations (Yang et al., 2004; Chaudhuri and Henderson, 2012). Its population is divided into four major phylogroups A, B1, B2, and D, and four rarer phylogroups C, E, F, and G that are differentially distributed among habitats (Clermont et al., 2015, 2019; Waters et al., 2020). Phylogroup A contained many of the human commensal strains. While B2 strains have characteristics that enabled better adaptation to the mammalian gut, they were found at higher proportion in wild animals than domesticated animals such as farm and zoo animals. On the other hand, strains that prevail in aquatic environments predominantly belong to phylogroup B1 (Ratajczak et al., 2010; Tenaillon et al., 2010; Berthe et al., 2013; Petit et al., 2017; Touchon et al., 2020).

Habitat-specific conditions such as the degree of anthropogenic influences have significantly shaped *E. coli*’s diversity and population structure by selecting for particular phenotypes and genotypes (Walk et al., 2007; Tenaillon et al., 2010; Van Elsas et al., 2011; Perchec-Merien and Lewis, 2013). For example, the widespread utilization of antibiotics in clinical and agricultural settings has enriched *E. coli* strains carrying class 1 integrons in human and animal hosts and also in external environments that are impacted by human activities (wastewater discharge, agriculture runoff, etc.) (Gillings et al., 2015). Owing to the fact that *E. coli* is used as a fecal pollution indicator bacteria, the vast majority of the studies on the genetic characteristics of the species have focused on environments that are impacted by human activities. Sites that are relatively undisturbed have received much less attention (Dusek et al., 2018). However, previous studies have shown that less disturbed sites were more likely to harbor novel genetically distinct strains of *E. coli* (Bermúdez and Hazen, 1988; Dusek et al., 2018). Extensive genome-based analyses suggested that the full genetic diversity and population structure of *E. coli* are indeed yet to be resolved entirely (Gonzalez-Alba et al., 2019; Touchon et al., 2020).

Population studies of *E. coli* became more perplexed with the discovery of the cryptic clades I to VI in *Escherichia* (Walk et al., 2009; Clermont et al., 2011; Gangiredla et al., 2018). These cryptic lineages are often misidentified as *E. coli* due to their resemblance to *E. coli* in many phenotypic and genetic characters (Cohan and Kopac, 2011; Deng et al., 2014; Walk, 2015). However, the cryptic lineages are in fact genetically divergent from *E. coli sensu stricto* and many are ecologically distinct from host-associated *E. coli* due to historical habitat differences. The members of clades III to V display characteristics of *ex vivo* adaptation, including higher prevalence in aquatic environments, low virulence and antibiotic resistance, poor GI-colonizing ability (except clade V), but better biofilm-forming ability than host-associated *Escherichia*. Contrarily, clade I, which is regarded as a subspecies of *E. coli*, appeared to be primarily host-associated, potentially pathogenic, and more resistant to antibiotics (Ingle et al., 2011; Luo et al., 2011; Perchec-Merien and Lewis, 2013; Deng et al., 2014; Quero et al., 2015; Vignaroli et al., 2015; Walk, 2015; Petit et al., 2017). These studies unraveled notable variations in the physiology, ecology, and clinical implications of these lineages as they establish different lifestyles. Such characteristics remained unknown for clade II because they remain rare despite thousands of host and non-host *E. coli*-like isolates had been screened in previous studies (Walk et al., 2009; Clermont et al., 2011; Lescat et al., 2013; Walk, 2015). Clade VI has been recently discovered, represented by an isolate that originated from a dog (Gangiredla et al., 2018).

The discovery of the cryptic clades and their primary association with non-host habitats (members of clades III to V specifically) reflected fragmentary comprehension of the evolution histories and ecology of *Escherichia* as a result of the under-sampling of external environments (Walk, 2015). More efforts to genetically characterize populations in understudied non-host habitats are required to obtain a holistic understanding of the biodiversity of *E. coli* and the cryptic clades. Examples are the tropical and subtropical aquatic environments where resources, stresses, and biotic factors are different from those in the temperate regions, in which the majority of relevant studies were conducted (Quero et al., 2015; Rochelle-Newall et al., 2015). Therefore, it is plausible that the distribution and genetic diversity of *E. coli* and cryptic clades and their ecological differentiation in such environments are different from those observed in temperate regions. From a practical perspective, the amplitude to which the presence of cryptic clades will confound *E. coli*-based water quality monitoring of these aquatic environments also needs further investigations.

To fill the knowledge gap, we analyzed the DNA sequences of seven housekeeping genes in 708 isolates of *E. coli* and cryptic clades obtained from multiple locations with different habitats and levels of human impacts in subtropical Hong Kong, China. We aimed at determining the genetic diversity and population structures of *E. coli* and cryptic clades present in different habitats as well as the roles of recombination and the nature of selection in generating genetic variations, and the extent of linkage disequilibrium and genetic differentiation among populations. Furthermore, using a larger dataset containing strains from other studies for a more comprehensive representation of habitat diversity, we inferred the ecological differentiation within *E. coli* and between members of *Escherichia* using an ecological partitioning tool. Focusing on a small watershed with contrasting level of human impact, we then elucidated how human activities shaped the genetic diversity and differential distribution of populations along this aquatic continuum. Ultimately, this study would reveal the diversity of *E. coli* and cryptic clades embedded in selected subtropical aquatic habitats, which will help achieve a more thorough understanding of the extent of their genetic and functional variations in relation to diverse habitats with varied conditions.

## Methodology

### Bacterial Isolate and Source of Isolation

This study analyzed 708 *E. coli*-like isolates recovered from samples collected from 18 locations around Hong Kong within the years 2008–2017. The isolates resembled typical *E. coli* (blue colonies) on CHROMagar™ ECC (CHROMagar Microbiology, Paris, France). Most isolates (*n* = 625) originated from water, periphyton, or sediment of freshwater and marine habitats. The remaining 83 isolates were of fecal origin (Table 1, Supplementary Figure 1, and Supplementary File 1). Reference strains (*n* = 105; 72 *E. coli* from ECOR collection, 30 clade I–VI, one each for *E. albertii*, *E. fergusonii*, and *Salmonella enterica*) were included for comparison whenever appropriate (Supplementary File 2).

**TABLE 1:** Source and sample type of the 708 isolates used in this study.

Sampling location	Number of isolate							Total
	Environmental			Fecal				
	Water	Periphyton	Sediment	Dog	Cow	Human*	Pig	
TK	163	122	96	–	–	–	–	381
KLH	1	–	196	–	21	–	–	218
Beach 1	22	–	–	–	–	–	–	22
Beach 2	7	–	–	–	–	–	–	7
Beach 3	5	–	–	–	–	–	–	5
Beach 4	4	–	–	–	–	–	–	4
Beach 5	3	–	–	–	–	–	–	3
Beach 6	3	–	–	–	–	–	–	3
Beach 7	1	–	–	–	–	–	–	1
Beach 8	1	–	–	–	–	–	–	1
Beach 9	1	–	–	–	–	–	–	1
Residential area 1	–	–	–	–	–	20	–	20
Residential area 2	–	–	–	–	–	14	–	14
Residential area 3	–	–	–	10	–	–	–	10
Residential area 4	–	–	–	9	–	–	–	9
Residential area 5	–	–	–	6	–	–	–	6
Residential area 6	–	–	–	2	–	–	–	2
Farm 1	–	–	–	–	–	–	1	1
TOTAL	211	122	292	27	21	34	1	708
	625			83				

*Sewage sample.

Most environmental isolates were from two locations: TK, a small freshwater watershed connected to a beach, and KLH, an intertidal mudflat (Supplementary File 1: detailed site description and sampling campaign). The remaining environmental isolates were from water samples randomly collected from nine beaches in 2014. For the TK watershed (381 isolates), 258 isolates (from water, periphyton, and sediment) were collected during the dry season of 2016 from 13 sites that spanned a gradient of human impact, from relatively undisturbed upstream catchwater and streams to downstream more impacted village and beach (low impact, 103 isolates; high impact, 155 isolates). This subset was further investigated for the influence of human activities on the distribution of clade II and *E. coli*. Another 123 isolates (39 from a storm outfall and 84 from the beach) were obtained from water samples of a follow-up investigation during the wet season of 2017.

The distinction of human impact level at the TK watershed was made based on on-site observations, such as distance from housing, beach activities, and human and vehicle traffic (Supplementary Figure 2 and Supplementary File 1). Six low human impact sites were situated upstream along the edge of a country park. Five of these sites were in three streams; the remaining site is a roadside drain receiving water from the country park located uphill. Seven high human impact sites were located downhill. One is a catchwater of stormwater that was channeled from uphill, situated in the vicinity of human settlement, including village houses and squatters, north of the TK village located downhill. Another three were two stormwater outfalls and a surface channel in the TK village, which is located next to the TK beach. The stormwater outfalls discharge directly into the beach water (three sampling sites). The beach area was perceived to receive the highest level of human disturbance due to beach activities, effluent from the Stonecutters Island Sewage Treatment Work, the largest sewage treatment plant in Hong Kong, and possible leakage from failing sewage infrastructure or cross-connected sewage and stormwater networks.

For KLH, a triangle-shaped, semi-enclosed intertidal mudflat, isolates were recovered from sediment at a single site, repeatedly sampled over 8 months spanning May 2009 to October 2010. The seaward margin of the mudflat faces north while a hill bounds its southeast margin and its southwestern margin bordered shrubland that is adjacent to a hill. Twenty-two houses were scattered around the shrubland, with the house closest to the mudflat being at least 100 m away. The mudflat is a sink for terrestrial runoff in the wet season due to the steep surrounding terrains. Observed fecal sources at the sampling site included a herd of approximately 15–20 feral cows and a few pet dogs. The maximum water depth of the mudflat is less than 2 m during high tides, whereas the sediment is fully exposed during low tides. The KLH isolates were screened with repetitive element palindromic genotyping to maximize genetic diversity while reducing sample size (see Supplementary Figure 3 and Supplementary File 1). Therefore, analyses that included the number of isolates (for example, frequency of genotype) shall be treated cautiously when the KLH dataset is included.

### Multilocus Sequence Typing

DNA was extracted using crude cell lysate method, followed by polymerase chain reaction (PCR) of the Achtman MLST scheme (Wirth et al., 2006) targeting seven loci: *adk* (adenylate kinase), *fumC* (fumarate hydratase), *gyrB* (DNA gyrase), *icd* (isocitrate/isopropylmalate dehydrogenase), *mdh* (malate dehydrogenase), *purA* (adenylosuccinate dehydrogenase), and *recA* (ATP/GTP binding motif).

The nucleotide sequences of amplicons were obtained through Sanger sequencing. Sequences were checked for quality, assembled, and trimmed using the Multilocus Sequence Typing (MLST) script in BioNumerics v7.1 (Applied Maths, Kortrijk, Belgium). Sequence-based and allele-based analyses were performed using the resulting sequences. For the allele-based approach, each distinct sequence at a locus represents a unique allele and was assigned an arbitrary allelic number. Each distinct combination of seven allelic numbers gives rise to a unique allelic profile (MLST genotype) denoted as sequence type (ST). Allelic and ST number was assigned using EnteroBase^1^ (Alikhan et al., 2018). Novel sequences were assigned new allelic numbers and STs locally.

### Phylogenetic Analysis

Phylogeny of the isolates was inferred by a sequence-based approach. Concatenated sequences of 3,423 bp each (*adk*-*fumC*-*gyrB*-*icd*-*mdh*-*purA*-*recA*) were generated for all isolates and reference strains. Resultant sequences were loaded into MEGA7 software v7 (Kumar et al., 2016) to build a multiple sequence alignment, followed by construction of a maximum likelihood (ML) tree (general time reversible G + I model, 500 bootstrap replications) and calculation of average evolutionary divergence (*p*-distance). *S. enterica* Typhimurium LT2 was used as outgroup to root the tree. The tree was used in the subsequent AdaptML analysis. Another simplified tree was built with the same parameters, but with each sequence type represented only once for the isolates.

### AdaptML Analysis

The AdaptML algorithms were used to investigate ecological differentiation within *Escherichia* in relation to their habitats (Hunt et al., 2008). An ML tree (generated as in the phylogenetic analysis section, from the concatenated MLST sequences of 708 isolates and 105 references) and sample sources were used as inputs for analysis. Isolates were categorized into 11 sample sources from human, animal (wild, domesticated, livestock, captive, avian, and unknown), and environments (freshwater, marine, freshwater marine mix, and unknown). While no further demarcations were made for human hosts, animal hosts were further differentiated. The animal hosts were mammalians and avian. The mammals were divided into five categories based on different lifestyles that affect the general likelihood of exposure to anthropogenic influences: livestock, domesticated, captive (zoo), wild, and unknown (lifestyles unknown or stated only as mammals). Environmental sources were further divided into four categories mainly based on salinity, i.e., freshwater, marine, freshwater marine mix (freshwater with occasional seawater intrusion), and unknown (for other environments, i.e., soil and where sources were only stated as environment or water). Default AdaptML parameters were used. ML tree overlaid with inferred ecological differentiation and metadata (phylogeny and sample sources) was visualized using iTOL (Letunic and Bork, 2019).

### Population Structure

Population structure was determined by allele-based approach. Single locus variants (SLVs), i.e., ST pairs sharing 6 alleles, were identified and assigned into clonal complexes (CCs) using geoBURST algorithm (Francisco et al., 2009) as implemented in PHYLOViZ v2.0 (Nascimento et al., 2016). A CC contained at least three STs, and its evolutionary descent patterns were inferred by identifying a founding genotype and its SLVs. Minimum spanning tree (MST) based on allelic profiles was generated using BioNumerics v7.1 (Applied Maths, Kortrijk, Belgium) and PHYLOViZ v2.0 (Nascimento et al., 2016).

### Population Genetics

The extent of recombination was visualized with a splits network using the NeighborNet algorithm (uncorrected *p* distance) in Splits Tree4 v4.14.6 (Huson, 1998; Huson and Bryant, 2005). The network was overlaid with *E. coli* phylogroup information. The assignment of phylogroup was done based on the correspondence between ST and phylogroup information in literature and MLST database. An *E. coli* ST was assigned into one of eight phylogroups according to information in Clermont et al. (2015, 2019) and EnteroBase (Alikhan et al., 2018). When phylogroup information is not readily available for a particular ST, its phylogroup was inferred by its genetic relatedness with STs of known phylogroup. Two STs were assumed to belong to the same phylogroup only if they are SLVs or double locus variants (DLVs). No assignment was made if criteria were not met. Subsequently the phi test (Bruen et al., 2006) implemented in the same program was used to test statistically for the role of recombination in generating (a) allelic variation at each locus (within gene recombination), and (b) different STs (within gene recombination + assortive recombination).

To determine if significant linkage disequilibrium (non-random association of alleles at loci) is present, the standardized index of association (I*^S^*A) was calculated (Haubold and Hudson, 2000), followed by the null hypothesis testing of linkage equilibrium in START2 (Jolley et al., 2001). Expected value for I*^S^*A is zero when alleles are in linkage equilibrium. A reduced dataset (each ST represented only once) was analyzed to minimize linkage disequilibrium introduced by sampling bias and/or possible existence of adaptive clones (Maynard Smith et al., 1993).

The nature of selection in generating allelic variation in each locus was assessed using the d_*N*_/d_*S*_ ratio by comparing the percentage of non-synonymous substitutions per non-synonymous site (d_*N*_) relative to synonymous substitutions per synonymous site (d_*S*_) (Nei and Kumar, 2000). Analysis was performed using START2 (Jolley et al., 2001). A d_*N*_/d_*S*_ ratio of 1 indicated neutral selection, < 1 suggested purifying selection, whereas > 1 implied adaptive or diversifying selection.

The fixation index, F_*ST*_, was calculated to assess pairwise genetic differentiation between different populations using Arlequin v3.0 (Excoffier et al., 2005). The lower the F_*ST*_ value, the higher the genetic similarity of two populations, indicating more gene flow.

### Detection of Class 1 Integron-Integrase (*intI1*) Gene

Clinical variant of the *intI1* gene was detected using PCR with the primer pair IntI1F165/IntI1R476 as described by Gillings et al. (2015). The assay was performed on the 258 isolates collected from the TK watershed during the dry season of 2016.

### Statistical Analysis

Statistical analyses were performed using the Minitab v18.1 software (Minitab Inc., Pennsylvania, United States).

## Results

### Phylogenetic Analysis

**FIGURE 1:**
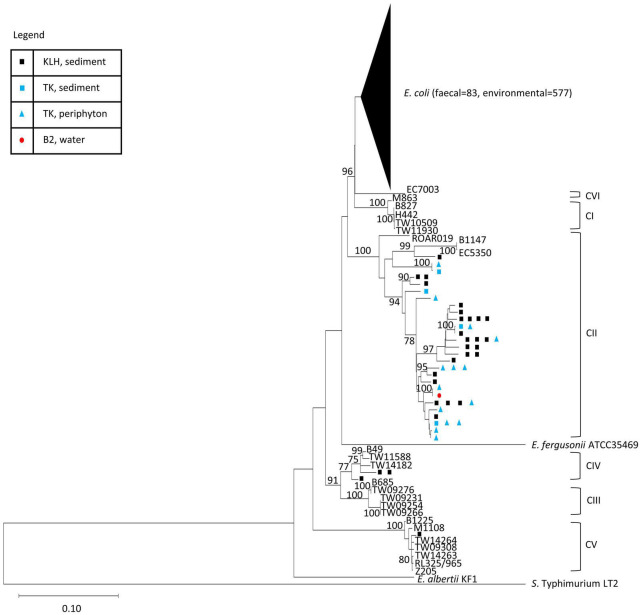
Phylogenetic position of the isolates in study in reference to strains of *Escherichia* spp. reported in previous studies. ML tree was built using concatenated sequences of seven MLST loci from the isolates and reference strains (general time reversible G + I model, 500 bootstrap replications). Bootstrap supports > 75% are indicated at the nodes. The genetic cluster containing all *E. coli* was collapsed (size of triangle not proportionate to number of entries) for ease of visualization of the whole tree. Each sequence type of the isolates was represented once. *S. enterica* Typhimurium LT2 was used as an outgroup and the reference strains for the cryptic clades were indicated. Complete tree containing all entries (708 isolates and 105 references) were presented in Figure 2. The isolation sources of the cryptic clades in this study were indicated at their respective terminal nodes.

The phylogeny of the isolates was established using an ML tree generated from the concatenated sequences of seven MLST loci. The resulting tree constituted several highly supported clusters (> 75% bootstrap support), which corresponded to known groups of *Escherichia* (Figure 1). Among the 708 isolates, 660 isolates (93%) constituted a major cluster with all reference *E. coli* from the ECOR collection (Ochman and Selander, 1984), clades I and VI with high bootstrap support (96%). The remaining 48 isolates (7%) formed monophyletic clusters with the reference strains of cryptic clades II, IV, and V, respectively. The majority of the cryptic clade isolates (*n* = 44) were associated with clade II, forming a highly supported (100%) cluster with three references of the clade, namely, ROAR019, B1147, and EC5350. The remaining four isolates belonged to clade IV (*n* = 3) and clade V (*n* = 1).

**FIGURE 2:**
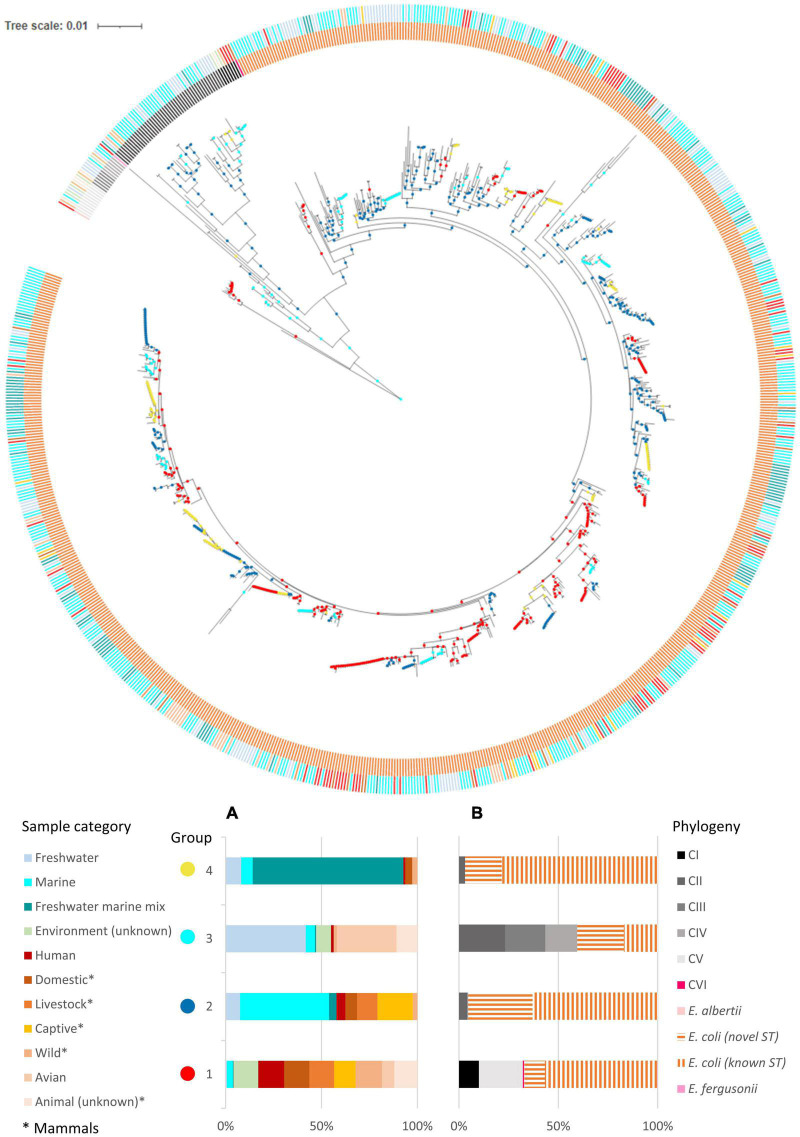
Inferred habitat associations of ecologically distinct groups of *Escherichia*. Metadata were mapped onto the ML tree built using concatenated sequences of seven MLST loci (general time reversible G + I model, 500 bootstrap replications). The outer ring indicated the sample category of the isolates while the inner ring indicated their phylogeny. Inferred ecologically distinct groups are shown as colored circles at the nodes. **(A)** Inferred ecologically distinct groups, defined by distinct distributions of isolates over the sample sources. **(B)** Distribution of phylogeny for each inferred group. All distributions are normalized by the total counts in each sample source to account for uneven sampling across sample categories.

All fecal isolates were *E. coli sensu stricto*, whereas environmental isolates consisted of both *E. coli* and cryptic clades. The cryptic clades (*n* = 48, 8% of the 625 environmental isolates) were mainly recovered from KLH and TK, except for a clade II isolate (from seawater of Beach 2). Clade II (*n* = 25) was the most abundant cryptic clades recovered from KLH, followed by clade IV (*n* = 3) and clade V (*n* = 1). Only clade II (100%, *n* = 18) was isolated from the TK watershed, during the dry season in 2016.

The average nucleotide divergence within group (*p*-distance) was 1.40% for *E. coli*, 0.59% for clade I, 2.22% for clade II, 0.73% for clade III, 0.94% for clade IV, and 0.27% for clade V. Notably, not only was the divergence within clade II higher than the other groups, the divergence between the clade II references alone (ROAR019 vs. B1147 and EC5350) was 3.7%. On the other hand, the nucleotide divergence between each pair of known sister clades was 2.6% (*E. coli* vs. clade I) and 2.3% (clade III vs. clade IV). These observations suggested finer classification of clade II into subclades similar to the above sister clades. However, confirmation of distinct subclades requires taxonomic analyses with higher resolution such as DNA–DNA hybridization and average nucleotide identity (Chun et al., 2018), and some of the branches within the clade II cluster lacked bootstrap support (< 75%). Therefore, we investigated the clade II-associated group in its entirety while recognizing the possible existence of subgroups.

### Habitat Prediction by AdaptML

We analyzed our data with the AdaptML algorithms (Hunt et al., 2008) to probe the ecological differentiation within *Escherichia*. The primary ecology questions under consideration were whether the members of *Escherichia* were ecologically differentiated in relation to their habitats (represented by different sample sources), in particular whether clade II was ecologically distinct from *E. coli*. From a dataset that contained STs from multiple sources spanning different hosts and environments, AdaptML inferred four ecologically distinct groups with different spectra of habitat associations (Figure 2). Each of these groups (namely, 1, 2, 3, and 4) is composed of sequence types (STs) with common characteristics in relation to their habitats.

The habitat spectra of groups 1 and 4 suggested clear ecological specialization. Group 1 reflected a primarily host-associated lifestyle, composed almost exclusively of *E. coli*, *E. albertii*, *E. fergusonii*, CI, and CVI STs isolated from diverse hosts and geographical locations. Clade V STs (originated from both hosts and environment), which were suggested to be capable of dual lifestyle in both host and environment (Vignaroli et al., 2015), were also included in this group. Group 4, on the other hand, is conspicuously enriched with isolates that could survive in a freshwater environment that was under occasional seawater intrusion. Majority of the group 4 STs were from the TK watershed, especially from sites encountering seawater intrusion (Supplementary Figure 4 and Supplementary File 3). Notably, group 4 contained only isolates from this study. Majority of the STs were *E. coli* while few clade II were present.

The habitat spectra of groups 2 and 3 suggested a more cosmopolitan characteristic, as both groups contained roughly an equal mixture of STs from both environment and hosts. Nonetheless, their constituent STs and habitats were markedly different. Group 2 contained mostly isolates from marine environments and animal hosts that are domesticated, captive (zoo animals), or grown as livestock. Similar to group 4, the isolates were almost exclusively *E. coli*, with few clade II present. On the other hand, unlike the other groups that contained predominantly *E. coli*, group 3 contained roughly an equal portion of *E. coli*, CII, CIII, and CIV. These isolates were mainly of freshwater and avian origins. Notably, STs of animal origins belonged to CII, CIII, and CIV. Majority of CII STs were contained in group 3. Also, while novel *E. coli* STs can be found in all groups (constituted 10, 32, 23, and 18% of their respective group, from group 1 to 4), slightly more than half (58%) of the *E. coli* STs in group 3 were novel. Many of these novel *E. coli* STs originated from the freshwater sites of the TK watershed, which remained largely undisturbed by human activities. Contrarily, most *E. coli* STs in the other three groups were known STs previously reported in the EnteroBase (Alikhan et al., 2018), which constituted 85% (group 1), 66% (group 2), and 81% (group 4) of the *E. coli* STs in their respective group. Our results thus demonstrated ecological differentiation within *E. coli* that were isolated from diverse habitats, and that in general clade II STs shared ecological similarities with only a small fraction of *E. coli*, many of which were newly described STs.

### Population Structure

In total, 380 STs were detected out of the 708 isolates (Table 2). *E. coli* (*n* = 660) contributed to 350 STs, 318 STs by *E. coli* from environmental source (*n* = 577) and 53 STs by fecal isolates (*n* = 83), with 21 common STs shared by both sources. Another 30 STs (44 clade II isolates contributed 27 STs; 3 clade IV, 2 STs; and 1 clade V, 1 ST) were contributed by the 48 cryptic clade isolates.

**TABLE 2:** Number of STs contributed by *E. coli* and cryptic clades.

	Environmental		Fecal		Total	
	(Isolate)	(ST)	(Isolate)	(ST)	(Isolate)	(ST)
*E. coli*	577	318	83	53	660	350
CI	0	0	0	0	0	0
CII	44	27	0	0	44	27
CIII	0	0	0	0	0	0
CIV	3	2	0	0	3	2
CV	1	1	0	0	1	1
CVI	0	0	0	0	0	0
Total	625	348	83	53	708	380

**FIGURE 3:**
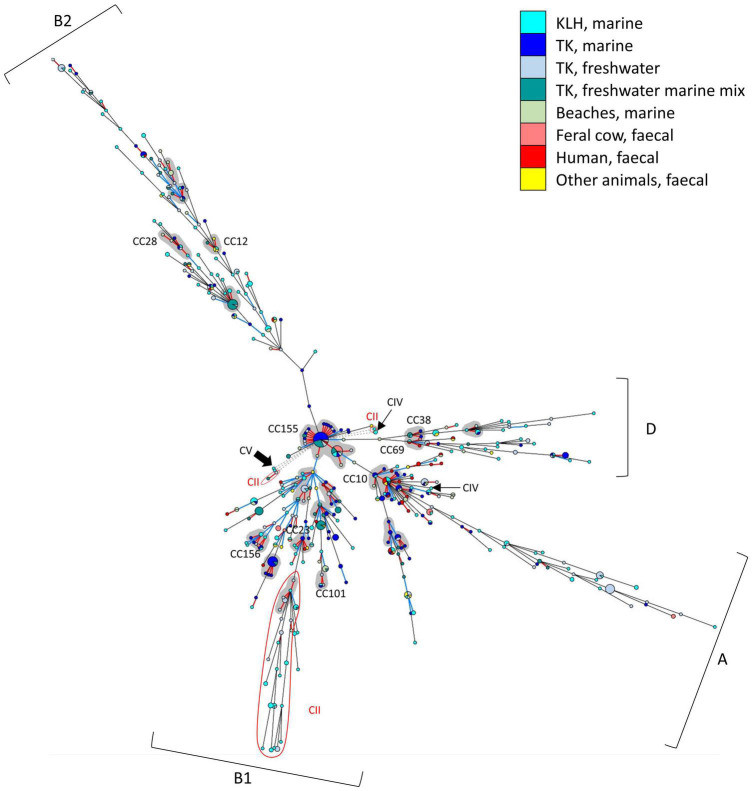
Minimum spanning tree (MST) displaying the genetic relatedness of the 708 isolates. Each node represented one ST. Node size is proportionate to the number of isolates contained in the node. Branch length is proportionate to distance between nodes, with red branch denoted 1 locus difference; blue: 2 loci; black: 3–6 loci; and gray dashed branch: no loci in common. CCs were shaded with gray background and nine well-established *E. coli* CCs that could also be identified in this study were labeled. *E. coli* phylogroups (A,B1,B2,D) for majority of the STs in each of the four main branches were indicated. The cryptic clade II STs were highlighted and indicated with red fonts; clade IV STs were indicated with thin black arrows; and a thick arrow indicated the sole clade V in this study.

The population structure of all 708 isolates was visualized using allele-based MST (Figure 3). MST revealed a largely clonal *E. coli* population structure containing a mixture of closely related STs (CCs, SLVs, and DLVs) and distant STs. *E. coli* of same phylogroup (mainly A, B1, B2, or D) generally clustered together, forming four main branches on the MST (see Supplementary Figure 6 and Supplementary File 4 for the same MST overlaid with phylogroup information). No clear demarcation of isolates according to geographical locations, sampling period, or sample types was observed, as many animal, human, and aquatic environment isolates of different geographical and temporal origins shared identical STs or were SLVs and DLVs.

Among *E. coli*, 153 STs (44%) very closely related, manifested as SLVs among which 21 CCs (containing 97 STs, 28% of all *E. coli* STs) were identified. Nine *E. coli* CCs were well-established CCs in EnteroBase (Alikhan et al., 2018), namely, CC10, CC12, CC23, CC28, CC38, CC69, CC101, CC155, and CC156. The two largest CCs in our dataset were CC155 (18 STs) and CC10 (12 STs), containing both fecal and environmental *E. coli* of different geographical and temporal origins. *E. coli* belonging to CCs of public health importance such as CC10, CC28, CC69, CC73, CC95, CC131, and CC155 (Riley, 2014; Skurnik et al., 2015; Stoesser et al., 2016; Reid et al., 2018) were present in both marine and freshwater habitats.

The clade II population was also clonal; 11 STs (41%) were SLVs and one CC was present, containing isolates mainly from the freshwater sites of TK (5 STs, 19%). The main clade II cluster was manifested as STs distantly related to *E. coli* phylogroup B1. The connecting clade II ST shared a common allele at *recA* (allele 7) with *E. coli* ST. Some clade II isolates from KLH, TK, and Beach 2 shared identical STs or were SLVs despite being isolated from different sample types and geographical origins. Majority of remaining STs exhibited ≥ 5 loci difference with each other.

A few STs were very distantly related to the rest of the isolates, without any allele in common. These STs included one *E. coli* ST, a pair of clade II SLVs, a clade II ST, one clade IV ST, and the sole clade V in our collection. The remaining clade IV ST shared a common allele with *E. coli* ST10 at *recA* (allele 2).

### Genetic Diversity and Differentiation of Different Populations

After looking at the expansive population structure of *E. coli* and clade II in their entirety, analyses were done at different population levels to yield ecologically meaningful comparisons. Populations were stratified into different levels (i.e., from the entire collection to subpopulations according to phylogeny, sampling dimensions such as host vs. environment, TK vs. KLH) to demarcate patterns of genetic variation, purifying selection strength, linkage disequilibrium, recombination, and genetic differentiation at/between different levels.

#### Sequence Type and Allelic Variation

Forty-five percent (*n* = 172) of STs were known STs available at EnteroBase (Alikhan et al., 2018) while the remaining 55% (*n* = 208) were novel (Table 3). New STs were significantly more abundant among environmental isolates than among fecal isolates (58 and 15%, respectively, *p* < 0.001). Number of alleles ranged from 74 for *purA* (frequency = 0.1) to 130 for *fumC* (frequency = 0.18) for all isolates.

**TABLE 3:** ST and allelic variation of data subset.

Subset (*n*)	ST		*adk*		*fumC*		*gyrB*		*icd*		*mdh*		*purA*		*recA*	
	*n* (f)	% new	*n* (f)	% new	*n* (f)	% new	*n* (f)	% new	*n* (f)	% new	*n* (f)	% new	*n* (f)	% new	*n* (f)	% new
All isolates (708)	380 (0.54)	55	119 (0.17)	41	129 (0.18)	36	117 (0.17)	37	120 (0.17)	45	75 (0.11)	32	74 (0.10)	32	83 (0.12)	31
All *E. coli* (660)	350 (0.53)	51	98 (0.15)	31	107 (0.16)	24	101 (0.15)	29	97 (0.15)	35	60 (0.09)	20	55 (0.08)	16	65 (0.10)	14
Fecal *E. coli* (83)	53 (0.64)	15	19 (0.23)	0	23 (0.28)	4	26 (0.31)	8	21 (0.25)	0	16 (0.19)	6	19 (0.23)	0	14 (0.17)	0
Environmental *E. coli* (577)	318 (0.55)	54	98 (0.17)	31	103 (0.18)	25	94 (0.16)	29	93 (0.16)	37	59 (0.10)	19	49 (0.08)	18	64 (0.11)	14
*E. coli* KLH (168)	138 (0.82)	43	55 (0.33)	15	69 (0.41)	23	66 (0.39)	15	55 (0.33)	18	44 (0.26)	9	37 (0.22)	11	49 (0.29)	14
*E. coli* TK (363)	181 (0.50)	56	58 (0.16)	33	60 (0.17)	17	62 (0.17)	24	68 (0.19)	38	36 (0.10)	17	32 (0.09)	19	42 (0.12)	5
Cryptic clades (48)	30 (0.63)	100	21 (0.44)	90	22 (0.46)	91	16 (0.33)	88	23 (0.48)	87	15 (0.31)	80	20 (0.42)	75	20 (0.42)	85
Cryptic clade II (44)	27 (0.61)	100	18 (0.41)	100	19 (0.43)	100	13 (0.30)	100	20 (0.45)	100	12 (0.27)	100	17 (0.39)	88	17 (0.39)	94
Cryptic clade II KLH (25)	15 (0.60)	100	12 (0.48)	100	15 (0.60)	100	9 (0.36)	100	13 (0.52)	100	9 (0.36)	100	14 (0.56)	93	12 (0.48)	100
Cryptic clade II TK (18)	13 (0.72)	100	8 (0.44)	100	8 (0.44)	100	9 (0.50)	100	10 (0.56)	100	4 (0.22)	100	7 (0.39)	71	8 (0.44)	88

*n, number of isolates; STs, or alleles. f, frequency, obtained by dividing the number of STs or alleles over the number of isolates. % new: percentage of STs or alleles that are novel.*

Considering only *E. coli*, the percentage of new STs among environmental isolates remained significantly higher than that of fecal isolates (54 vs. 15%, *p* < 0.05) (Table 3). Moreover, environmental *E. coli* isolates noticeably displayed more new alleles for all loci (from 14% for *recA* to 37% for *icd*) than fecal isolates (from 0% for *adk*, *icd*, *purA*, and *recA* to 8% for *gyrB*). However, environmental isolates have fewer alleles across all seven loci than fecal isolates despite contributing more new alleles. *E. coli* generally has lower diversity for *mdh*, *purA*, and *recA*.

All 30 cryptic clade STs were newly discovered. Most were distantly related to that of the reference cryptic clades (≤ 1 allele in common), except for the sole clade V isolate, which is a DLV of ST2721 (ST of reference clade V strain E1118). All clade II isolates have no common allele with the reference clade II STs (Supplementary Figure 5 and Supplementary File 4). For cryptic clades, at least 75% of alleles for each locus were new alleles. Clade II exhibited lowest diversity for *mdh*. Compared to *E. coli*, generally more allele per locus was observed for cryptic clades collectively and for clade II specifically.

#### Nature of Selection

A low d_*N*_/d_*S*_ value was observed for all loci, indicating strong purifying selection acting at these loci to limit amino acid polymorphisms (Table 4). Thus, allelic variations at the housekeeping loci were mainly contributed by silent mutation or recombination. Different strength of purifying selection was observed across the seven loci, with recA under strongest purifying selection across all subsets.

**TABLE 4:** d_N_/d_S_ ratio of the seven housekeeping genes.

Subset	*n*	*adk*	*fumC*	*gyrB*	*icd*	*mdh*	*purA*	*recA*
All isolates	708	0.0137	0.0168	0.0513	0.0338	0.0187	0.0376	0.0018
All *E. coli*	660	0.0167	0.0202	0.0374	0.0475	0.0342	0.0357	0.0041
Fecal *E. coli*	83	0.0127	0.0179	0.0258	0.0092	0.0424	0.0071	0.0000
Environmental *E. coli*	577	0.0167	0.0202	0.0372	0.0490	0.0302	0.0380	0.0042
*E. coli* KLH	168	0.0139	0.0162	0.0246	0.0060	0.0209	0.0074	0.0056
*E. coli* TK	363	0.0160	0.0222	0.0306	0.0633	0.0150	0.0549	0.0000
Cryptic clades	48	0.0108	0.0198	0.0247	0.0022	0.0154	0.0478	0.0000
Cryptic clade II	44	0.0173	0.0231	0.0367	0.0032	0.0131	0.0373	0.0000
Cryptic clade II KLH	25	0.0062	0.0244	0.0195	0.0000	0.0000	0.0375	0.0000
Cryptic clade II TK	18	0.0266	0.0215	0.0439	0.0064	0.0290	0.0262	0.0000

For *E. coli*, fecal isolates showed ∼5-fold lower d_*N*_/d_*S*_ ratios for *icd* and *purA* than environmental isolates. Among environmental *E. coli*, d_*N*_/d_*S*_ for *icd* and *purA* were ∼10- and 7-fold lower, respectively, for isolates from KLH than those from TK. Within clade II, isolates from KLH generally underwent stronger purifying selection at more than half of the loci. Disparate d_*N*_/d_*S*_ were most evident at *icd* and *mdh* between isolates from TK and KLH. When comparing *E. coli* and clade II, d_*N*_/d_*S*_ for *icd* were more dissimilar, with clade II having ∼15-fold lower ratio. Specifically, comparing *E. coli* and clade II from KLH, which were co-isolated from a single site, clade II experienced stronger purifying selection for most loci except for *fumC* and *purA*.

#### Linkage Disequilibrium

Extensive linkage disequilibrium was detected (I*^S^*A > 0 and *p* = 00.00 for all subsets), indicating the presence of clonality in population structure at all tested levels (Table 5).

**TABLE 5:** Test of linkage equilibrium.

Subset	No. of isolate	I*^S^*A*	*p*
All isolates	708	0.2832	<0.001
All *E. coli*	660	0.2760	<0.001
Fecal *E. coli*	83	0.2608	<0.001
Environmental *E. coli*	577	0.2878	<0.001
*E. coli* KLH	168	0.2084	<0.001
*E. coli* TK	363	0.3556	<0.001
Cryptic clades	48	0.4879	<0.001
Cryptic clades II	44	0.4763	<0.001
Cryptic clade II KLH	25	0.5170	<0.001
Cryptic clade II TK	18	0.5286	<0.001

*1,000 trials.

#### Recombination

**FIGURE 4:**
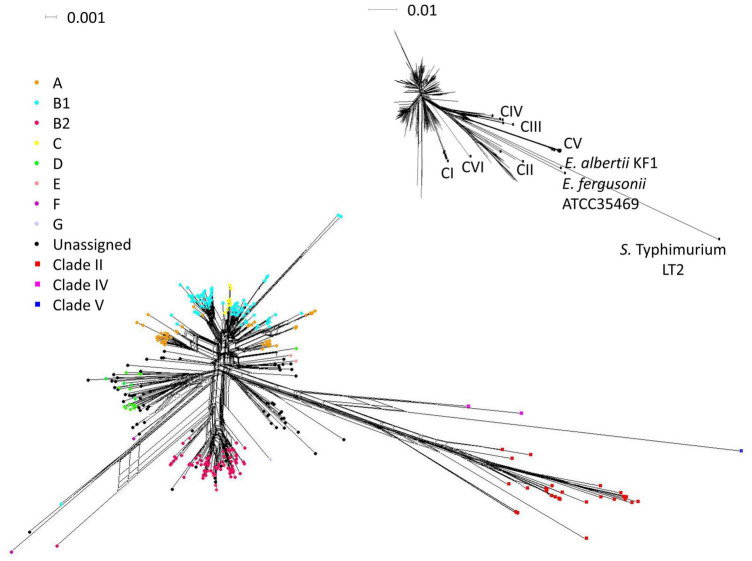
Phylogenetic network of the 380 STs based on NeighborNet algorithm using concatenated sequence. Parallelogram denotes incompatible partition within sequence data (conflicting phylogenetic signals due to recombination or recurrent mutation). Insert is the network generated using the same dataset plus reference sequences as used in Figure 2. References of cryptic clades I–VI, *E. albertii* KF1, *E. fergusonii* ATCC 35469, and *Salmonella enterica* Typhimurium LT2 were indicated.

Network analysis revealed that majority of *E. coli* STs clustered according to phylogroups, except for phylogroup A, which was split into two main clusters (Figure 4). The cryptic clades formed clusters distinct from *E. coli*. The network largely resembled a tree-like structure although parallelograms representing conflicting phylogenetic signals (mainly due to recombination) were present. Intra-phylogroup recombination was more evident, which contrasted limited inter-phylogroups recombination. Recombination was also evident within cryptic clades but essentially rare between cryptic clades and *E. coli*.

The phi test (Bruen et al., 2006) was performed to detect statistically significant recombination: within different *E. coli* populations, between *E. coli* and cryptic clades, within cryptic clades, and within clade II (Table 6). Recombination was significant in generating different STs for all subsets (*p* < 0.05). Generally, with cryptic clades included, different extent of recombination existed across all seven loci, with *adk* having the least recombination and *fumC* having the most extensive recombination. Recombination within *E. coli* and within cryptic clades was more significant compared to recombination between the two groups. Similarly, recombination was more significant within fecal *E. coli* and within environmental *E. coli* than between these two populations. Among *E. coli*, recombination was significant both within and between population subsets for *fumC* and *icd*, but was only significant within subpopulations for *mdh* and *purA*.

**TABLE 6:** Recombination analysis by phi test (Bruen et al., 2006) with *p-*values indicated.

Subset	*n*	*adk*	*fumC*	*gyrB*	*icd*	*mdh*	*purA*	*recA*	ST
All isolates	708	0.415	<0.050	0.962	<0.050	0.897	0.379	0.246	<0.050
All *E. coli*	660	0.963	<0.050	0.904	<0.050	0.081	0.122	0.138	<0.050
Fecal *E. coli*	83	0.618	<0.050	0.335	<0.050	<0.050	<0.050	0.473	<0.050
Environmental *E. coli*	577	0.944	<0.050	0.966	<0.050	<0.050	<0.050	0.081	<0.050
Environmental *E. coli* + cryptic clades	625	0.495	<0.050	0.980	<0.050	0.818	0.223	0.109	<0.050
Cryptic clades	48	0.252	<0.050	<0.050	<0.050	0.879	0.156	0.241	<0.050
Cryptic clades (including references)	76	<0.050	<0.050	<0.050	<0.050	0.990	<0.050	0.172	<0.050
Cryptic clade II	44	0.078	<0.050	<0.050	0.068	0.221	0.142	<0.050	<0.050
Cryptic clade II (including references)	46	0.099	<0.050	0.116	0.060	0.797	0.085	<0.050	<0.050
*E. coli* TK + clades II TK	381	0.726	<0.050	0.989	<0.050	<0.050	0.144	<0.050	<0.050
*E. coli* TK	363	0.980	<0.050	0.894	<0.050	<0.050	<0.050	0.519	<0.050
Cryptic clade II TK	18	0.262	<0.050	<0.050	<0.050	0.181	0.599	0.108	<0.050

*Significant evidence of recombination if p < 0.05.*

#### Genetic Differentiation

All pairwise F_*ST*_ values between different *E. coli* populations (same subsets as in Table 3) were less than 0.05000 (*p* < 0.05), indicating a lack of genetic differentiation between tested populations (Supplementary File 5). The environmental and fecal *E. coli* subsets only showed little genetic difference (*F*_*ST*_ = 0.01539, *p* < 0.01). On the other hand, gene flow between the cryptic clades (collectively and clade II specifically) and *E. coli* were infrequent (*F*_*ST*_ > 0.5, *p* < 0.01). Moderate genetic difference existed between cryptic clade II from KLH and TK (*F*_*ST*_ = 0.09912, *p* < 0.01).

#### Distribution of *Escherichia coli* and Cryptic Clade II in a Watershed With Distinct Human Impact

To investigate the distribution of clade II and *E. coli* in relation to human impact, we further examined isolates that were collected from two differentially impacted areas in the TK watershed during the dry season of 2016. We examined the genetic differentiation of isolates from the two areas by first including both *E. coli* and clade II. Moderate genetic differentiation existed between the two groups of isolates under differential human impact (*F*_*ST*_ = 0.07031, *p* < 0.01). However, when clade II isolates were removed, little genetic differences were observed between the two groups of *E. coli sensu stricto* (*F*_*ST*_ = 0.04846, *p* < 0.01).

**FIGURE 5:**
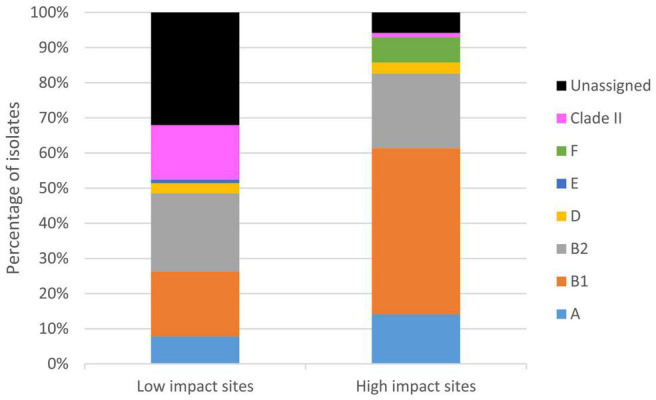
Distribution of *E. coli* phylogroups and clade II isolates in the TK watershed. An *E. coli* ST was assigned into one of the eight phylogroups according to information in Clermont et al. (2015, 2019) and EnteroBase (Alikhan et al., 2018). When information is not readily available, phylogroup was inferred for an ST by its genetic relatedness with STs of known phylogroup: two STs were assumed to belong to the same phylogroup only if they are SLVs or double locus variants (DLVs). No assignment was made if criteria were not met.

In fact, majority of the clade II isolates (89%, *n* = 16) were recovered from the low human impact sites. Prevalence of clade II isolates was significantly higher (*p* < 0.01) at the low human impact sites (16% of 103 isolates) than at the high human impact sites (1% of 155 isolates). Clade II isolates were present in 83% of periphyton and 40% of sediment samples collected from the low human impact sites, constituting 10–60% of analyzed isolates per sample when present. The two clade II isolates from high human impact sites were from periphyton and sediment of storm outfall 1 in TK Village, each constituting 3% of analyzed isolates of respective sample (Supplementary Table 1 and Supplementary File 1). Besides clade II, *E. coli* phylogroups were also differentially distributed in the two areas (Figure 5). Notably, the low impact sites contained more *E. coli* without phylogroup assignment.

Novel STs (of *E. coli* and clade II) were retrieved at a higher frequency from the low human impact sites than sites with higher impact (68 vs. 40%, *p* < 0.01), contributed by 69 and 22% of isolates, respectively (*p* < 0.01). Considering only *E. coli* (as all clade II STs were novel), the percentage of new STs from the low human impact sites remained noticeably higher (61 vs. 38%, *p* = 0.02; contributed by 63 and 21% of isolates, respectively, *p* < 0.01).

On the other hand, *E. coli* isolates belonging to CCs of public health concern (CC10, CC28, and CC155) were mainly found at high human impact sites (*n* = 28, *p* < 0.01). Only one such isolate was detected in the low human impact sites. All isolates were then screened for the integrase gene of class 1 integron (*intI1*), which was proposed as a marker for anthropogenic pollution (Gillings et al., 2015). Notably, all *intI1*-positive isolates were *E. coli*. The percentage of *intI1*-positive *E. coli* was higher at the high human impact sites (12%) than at the low impact sites (2%), and the differences were statistically significant (*p* = 0.01).

## Discussion

This study reported a relatively large number of clade II isolates from the less studied subtropical aquatic habitats, which, when present, constituted up to 60% of analyzed presumptive *E. coli* isolates per sample. In previous studies, clade II had been rarely observed despite thousands of host and non-host isolates resembling *E. coli* were screened, with only three fecal strains reported (Walk et al., 2009; Clermont et al., 2011; Lescat et al., 2013; Walk, 2015; Gangiredla et al., 2018). Two studies reported the presence of cryptic lineages (which included clade II) and *E. coli* in aquatic habitats, but each lineage’s proportions were not mentioned (Quero et al., 2015; Petit et al., 2017). Thus, little was known about the types of habitats where clade II may prevail, let alone other ecological characteristics. Clade II isolates were exclusively found in environmental matrices in this study, and none were from fecal samples. In contrast to most studies reporting that environmental cryptic clades belonged to clade V predominantly (see Supplementary File 6; Walk et al., 2009; Clermont et al., 2011; Deng et al., 2014; Vignaroli et al., 2015), here clade II was the most abundant from both freshwater and seawater habitats. Clade II accounted for all cryptic clades isolated at the primarily freshwater TK watershed and for 86% of cryptic isolates in the KLH mudflat (Figure 1).

We showed that periphyton and sediment were significant sources of clade II (Supplementary Table 1 and Supplementary Files 1, 6). Most clade II isolates (89%) were recovered from periphyton in the low human impact sites in the TK watershed. Despite a greater sampling effort, only two clade II isolates were recovered from the high human impact area downstream of the less disturbed area. Later, we obtained similar results in another study that was conducted at similar locations in the TK watershed (unpublished data). Cryptic clades (clade II = 25, clade IV = 3, clade V = 1) were also repeatedly isolated from coastal marine sediment over 8 months in the KLH mudflat, which was also a relatively undisturbed site, albeit having higher human impact level than the low impact sites in TK. Our high frequency of clade II isolation could be attributable to multiple factors. It is likely that pristine environments harbored more clade II, or that compared to temperate regions, subtropical environments generally sustained different proportions of cryptic lineages. Knowledge on the ecology of clade II will benefit from biogeography studies of the distribution of different cryptic clades in temperate, tropical, and subtropical aquatic environments and within diverse wildlife, which was less studied. Another pertinent possibility worth investigating is whether selective media have differential selectivity against different cryptic lineages. ChromAgar™ ECC was utilized in this study, whereas other studies utilized modified TEC, FC, modified FC, or RAPID’*E. coli* 2 Medium (Walk et al., 2007; Deng et al., 2014; Quero et al., 2015; Vignaroli et al., 2015; Petit et al., 2017). ChromAgar™ ECC was chosen in this study in accordance with the *E. coli* detection method developed by the Environmental Protection Department of Hong Kong for routine beach water quality assessment (Ho and Tam, 1997; Thoe et al., 2018).

Nonetheless, our observations that clade II prevail in subtropical aquatic environments with low human interference resonated another study where clades II, III, and V were abundant in river sediments that were less disturbed by human and agriculture activities (Petit et al., 2017). Such high frequencies of non-clade I cryptic clades could potentially confound the use of *E. coli* as fecal indicator bacteria. When they are abundant in sediments and associated vegetation, these cryptic clades could be suspended into overlaying water upon perturbations, for example, by storm events. Since current monitoring methods would misidentify them as *E. coli* unless MLST or PCR targeting cryptic clades (Clermont et al., 2011) were performed, this may cause a false conclusion of recent fecal pollution. Thus, it is imperative to determine their actual potential in interfering with *E. coli*-based water quality monitoring under different environmental perturbations or hydrology conditions.

While the observations above suggested that relatively undisturbed aquatic environments could be natural habitats of clade II, their presence there could have also possibly originated from recurring fecal input by wildlife, as the reference clade II strains originated from fecal materials of bird and yellow-backed duiker (Skurnik et al., 2006; Walk et al., 2009; Clermont et al., 2011; Gangiredla et al., 2018). Nonetheless, the absence of class 1 integrons in our clade II isolates was an indication that their habitat (be it wild animal host or external environment) was less disturbed by human activities. Integrons are genetic platforms capable of capturing and expressing gene cassettes that confer adaptive capabilities such as antimicrobial resistance. Class 1 integrons are the most clinically relevant, causing widespread dissemination of multidrug resistance (Cambray et al., 2010). Previously it was demonstrated that the prevalence of class 1 integrons in fecal *E. coli* was positively correlated with the exposure of their animal hosts to human activities (Skurnik et al., 2006) and that the integrase gene of class 1 integron could be a marker for anthropogenic pollution (Gillings et al., 2015).

Our hypothesis that the natural habitats of clade II were relatively pristine was supported by the AdaptML analysis (Figure 4), which inferred ecologically differentiated groups from phylogeny and ecological data (sample sources, which represented different types of habitats) using a hidden Markov model. Our dataset was heavily enriched with environmental isolates, while fecal isolates were underrepresented. Thus, to overcome the bias and for a more comprehensive representation of the habitats of *E. coli* and cryptic clades, reference strains from diverse sources were included in the analysis. The expanded dataset included strains representing the typical diversity of *E. coli* (Ochman and Selander, 1984) and representative genotypes of the cryptic clades. The analysis indicated that the ecological niche of clade II is distinct from typical host-associated *E. coli* and also cryptic clade I, V, and VI. Instead, most clade II genotypes shared habitats with the primarily environment-associated clades III and IV, and with *E. coli* that were mainly isolated from less disturbed freshwater habitats. The AdaptML prediction was conservative and inferred ecological differentiation based on our anticipated ecological categories (Hunt et al., 2008; Francisco et al., 2014). Therefore, these phylogenetically distinct groups may be further differentiated for other ecological parameters, as we focused on coarse dimensions such as host and environment types. Moreover, the use of merely seven housekeeping genes may miss out on ecological specializations that were not reflected in these genes. Sensitive and high-resolution approaches based on whole-genome data would further detail the ecological divergence between clade II and environmental *E. coli*, or even within clade II itself.

As clade II strains were found in wildlife and aquatic habitats, they could resemble clade V that displayed traits of dual lifestyle in both host and external environments (Vignaroli et al., 2015), or represented environment-associated lineages with wildlife as spillover hosts, or consisted of at least two distinct ecotypes (host- vs. environment-associated). While our clade II isolates and the reference clade II strains formed a monophyletic cluster (Figure 1), the cluster’s relatively high genetic divergence and subclustering indicated possible ecological divergence. Moreover, the host-associated reference strains were generally more distantly related to our isolates of environmental origins. However, biogeographic effects cannot be dismissed (Cohan and Perry, 2007; Hunt et al., 2008). While our isolates were from Hong Kong, China, the host-associated clade II strains originated from Australia, Gabon, and United States (Walk et al., 2009; Clermont et al., 2011; Lescat et al., 2013; Walk, 2015; Gangiredla et al., 2018). We previously demonstrated that two groups of clade II genomes displayed signatures of functional divergence that were reflective of ecological differentiation to occupy gastrointestinal and external environments, respectively (Shen et al., 2020). The study included genomes of isolates in this study. Combined, results from our studies suggested that some of the clade II isolates in this study were closely related to the original clade II strains of fecal origins but were ecologically differentiated. Comparative genome sequence analysis will allow in-depth inquiries of the further phylogenetic and ecological differentiation within clade II.

The population structure of clade II was a mixture of clonal and divergent genotypes (Figure 2). Clade II STs were generally more distantly related, even for isolates from the same sample. More isolates need to be analyzed to verify the observed patterns. Although cryptic clades coexist with *E. coli* in aquatic environments, we observed limited recombination of the housekeeping genes between *E. coli* and cryptic clades II, IV, and V in this study (Table 6). The large F_*ST*_ value (> 0.5) between clade II vs. different *E. coli* populations also indicated insignificant gene flow (Supplementary File 5). The sharing of alleles, however, is not absent. For example, the phylogenetically distinct *E. coli* and clade II shared a common allele at the *recA* locus (Figure 2). Likewise, comparative genomics showed that core gene recombinations were mainly restricted to within enteric *Escherichia* (including clade I) or within environment-associated clades III–V. However, clade II was not included in the analysis (Luo et al., 2011).

In this study, all seven MLST loci were under purifying selection, as anticipated for housekeeping genes (Chaudhuri and Henderson, 2012). Nonetheless, for the same gene, we observed different selection strengths between phylogenetically distinct *Escherichia* (*E. coli* vs. cryptic clade II) and for populations isolated from environments of different conditions (host-associated vs. environmental; primarily freshwater vs. marine), signifying different evolutionary processes among these compared entities (Table 4). Different populations are likely subjected to different selection pressures specific to particular environmental and ecological conditions (Feil and Spratt, 2001). In general, marine habitats exerted stronger selection on the housekeeping genes of both *E. coli* and clade II than freshwater habitats (Table 4). At the KLH mudflat, stronger purifying selection was exerted on clade II than *E. coli* for most of the genes. At this point, whether this is an exemplification of one group being better at coping with the marine environment remained debatable. Similarly, the selection strength exerted on clade II in host vs. external environment was not probed as there were merely three fecal isolates with genetic information. These questions could be better answered when more isolates from different habitats are available and analyzed with approaches that are more sensitive in detecting response to selection.

Thus far, this work revealed previously undescribed genetic diversity of clade II (all STs and most of the alleles at the majority of MLST loci were novel) and uncovered some of their ecological characteristics and population genetics. Similarly, many new STs and alleles were also found within environmental *E. coli*. The discovery of these new genetic diversities attested to the biodiversity potential of the subtropical aquatic environment. The isolates may represent more genetic diversity than was demonstrated, as the Achtman MLST scheme adopted in this study has the lowest nucleotide diversity among the three *E. coli* MLST schemes (Kaas et al., 2012). Nonetheless, the Achtman MLST scheme was chosen for its phylogeny based on concatenated sequence data have the best congruency with the “true” phylogeny represented by whole-genome data (Sahl et al., 2012).

For *E. coli*, we noticed that while the environmental isolates contributed significantly more novel STs and alleles than the fecal counterparts, they displayed lower ST frequency and fewer alleles across all seven loci (Table 3). In environmental habitats, *E. coli* need to survive conditions that differ substantially from hosts, such as sunlight radiation, nutrient limitation, high salinity (in marine habitat), and predation (Van Elsas et al., 2011). Thus, environmental *E. coli* population structure depends on both origin and environmental survivability: rapid decay, persistence, or naturalization (Van Elsas et al., 2011; Berthe et al., 2013). Moreover, the cultivation method may miss out on injured or dormant cells that were viable but non-culturable. Consequently, environmental populations typically were less diverse than host-associated populations despite having more novel genotypes (McLellan, 2004; Walk et al., 2007; Perchec-Merien and Lewis, 2013). Our observations above not only indicated a discovery of new diversities that suggested adaptation to specific environments (Perchec-Merien and Lewis, 2013), but also exemplified natural selection favoring genotypes that survive better in the external environment (McLellan, 2004; Walk et al., 2007; Perchec-Merien and Lewis, 2013).

This study observed a largely clonal population structure for *E. coli* (Figure 2), corroborating the global *E. coli* phylogeny (Tenaillon et al., 2010; Chaudhuri and Henderson, 2012). Clonality was implied by the presence of extensive linkage disequilibrium despite the significant role of recombination in generating allelic and ST variations at all population levels (Table 5). Strong linkage arose probably due to natural selection imposed by specific environmental conditions (Walk et al., 2007; Perchec-Merien and Lewis, 2013). Clonality was also indicated by isolates sharing identical STs or forming CCs despite diverse geographical and temporal origins (Figure 2). Similar observations were reported elsewhere (Maynard Smith et al., 1993; Wirth et al., 2006; Walk et al., 2007; Perchec-Merien and Lewis, 2013). The lack of geographical and temporal differentiation was further confirmed by little genetic differentiation between different *E. coli* populations, as reflected by low F_*ST*_ values (Supplementary File 5). All these observations are testaments to the clonality of *E. coli*. Intra-phylogroup recombination was more frequent while inter-phylogroup recombination was limited (Figure 3 and Table 6). These observations corroborated Walk et al. (2007), albeit utilizing different sets of housekeeping genes. Analysis with single-nucleotide polymorphisms also demonstrated that inter-phylogroup recombination was primarily between A and B1, A and E, and B2 and D, with ecological niche partitioning among plausible explanations (Leopold et al., 2011).

AdaptML analysis iterated the broad ecological niche of *E. coli*, with its genotypes distributed over all four ecologically distinct groups (Figure 4, Supplementary Figure 4, and Supplementary File 3). While group 1 consisted mainly of typical host-associated *E. coli*, group 2 appeared to lead a more generalist lifestyle. This group contained roughly equal portions of isolates from both host and environmental sources from various locations, reflecting a possibility that these genotypes not only were adapted to life within hosts but may also persist in the external environments, especially marine environments. Group 4 appeared to harbor characteristics that enabled survival in environments with occasional salinity fluctuation due to seawater intrusion. Finally, group 3 notably harbored many novel *E. coli* genotypes and grouped with most clade II genotypes. These novel genotypes were mainly from the TK freshwater sites where many clade II were isolated, which remained largely undisturbed by human activities. Thus, this group of *E. coli* may represent genotypes that were more suited to live in relatively pristine environments. Further research is warranted to confirm the ecological distinctness of these inferred groups and the underlying functional divergence that resulted in such differentiation. It is also investigation-worthy to determine if genotypes of groups 3 and 4 contained naturalized *E. coli*.

In this Anthropocene epoch, human activities have significantly altered the dynamics of microbes (and their gene repertoire) dissemination (Zhu et al., 2018). The TK watershed is an aquatic continuum with differential human impacts where human activities/land use shaped the population structures of *E. coli* and the distribution of clade II. The clade II isolates were the main contributor to the moderate genetic difference between populations (containing both *E. coli* and clade II) from the two differentially impacted areas. Little genetic differentiation was present when clade II isolates were removed, exemplifying the effect they could bring to *E. coli* population genetics study when present in high frequency and not properly distinguished.

Nonetheless, by looking at both *E. coli* and clade II, we were able to investigate the partitioning of these two *Escherichia* in relation to human activities. The low impact area harbored *E. coli* populations with a significantly higher proportion of novel genotypes than the high impact area. Moreover, clade II isolates were conspicuously abundant in sediment and periphyton of the low impact area (Supplementary Table 1 and Supplementary File 1). The differential composition of *E. coli* phylogroups between the two areas was not elaborated as the phylogroups for many isolates from the low impact sites (slightly over one-third) were unassigned (Figure 5). Nonetheless, the presence of this many isolates with unassigned phylogroups is a manifestation of the newly discovered diversity of *E. coli* from these sites, which were genetically more distant to known sequence variants in the public MLST database. All these observations pointed to less human fecal pollution at these sites. *E. coli* that were present likely originated from wild animal feces, and some could even represent genotypes that are more adapted to external environments.

Shifting downstream to the high human impact area, the abundance of clade II isolates and novel *E. coli* genotypes reduced substantially, and was contrasted by a significant increase of *E. coli* belonging to globally successful CCs of public health concern, such as CC10 and CC155. ST155 (primary founder of CC155, the largest CC in our study) has been suggested to be responsible for the spread of extended-spectrum β-lactamase gene from animals to humans, causing widespread resistance to β-lactam antibiotics (Skurnik et al., 2015). The second-largest CC in our dataset, CC10, was associated with multiple *E. coli* pathotypes, found in a wide range of hosts and environmental matrices, and frequently antibiotic-resistant (Reid et al., 2018). The concentration of these genotypes around the high human impact area suggested the directionality of the flow of these isolates from human activities. As selection was neutral or purifying in the slow-evolving housekeeping genes that encode essential functions (Zheng et al., 2004), detecting genes that respond quickly and positively would better depict how bacteria respond to selection pressures in habitats. Examples are mobile genetic elements such as prophages and integrons, which have been vital for habitat adaptation (Touchon et al., 2020). Class 1 integrons enabled *E. coli* to better adapt to antibiotic pressure with very low fitness cost and were not stably maintained without selection pressure (Díaz-Mejía et al., 2008; Lacotte et al., 2017). Thus, when these *E. coli* are introduced into receiving water contaminated with antibiotics, class 1 integrons are retained as the selection pressure calls for its maintenance and expression. Sources of antibiotic contamination include the discharge of antibiotic residues from sewage outfall, antibiotics runoff from animal farming on surrounding lands, and administration of antibiotics in aquaculture. The presence of class I integrons was influenced by human activities as shown in other studies (Gillings et al., 2015; Petit et al., 2017; Uyaguari-Díaz et al., 2018; Touchon et al., 2020). *intI1*-positive *E. coli* were more abundant at the high human impact sites that were within the vicinity of the beach. These sites received the highest level of human disturbance: direct body contact (beach activities), sewage effluent discharge, and possible leakage from failing sewage infrastructure or cross-connected sewage and stormwater networks. Meanwhile, as mentioned earlier, clade II isolates, which were mainly found in low impact sites, were all *intI1*-negative.

In conclusion, this study unearthed the vast genetic diversity of cryptic clades and *E. coli* embedded in the freshwater and marine habitats in Hong Kong, attesting to the less tapped biodiversity potential of these subtropical environments. Contrary to previous reports, clade II was the most abundant cryptic lineage co-isolated with *E. coli*, especially in relatively pristine subtropical aquatic environments. Clade II and *E. coli* showed limited recombination and significant genetic divergence. Our works provided preliminary indications that clade II was ecologically differentiated from typical *E. coli*. Some members may even represent novel environmental *Escherichia* clades closely related to the original clade II strains of fecal origins. Many questions remained about the ecological and clinical significance of clade II and their further phylogenetic and ecological differentiation. Nonetheless, discovering the habitats where clade II maintained relatively large population sizes and the availability of genome-based data would pave the ways to answer these questions. *E. coli* of diverse origins exhibited clonality amidst divergent STs, echoing other studies in that recombination in housekeeping genes was insufficient to disrupt phylogenetic signals of the largely clonal *E. coli*. Notably, environmental *E. coli* were less diverse than fecal isolates despite contributing many new alleles and STs. Finally, we demonstrated that human activities influenced *E. coli* and clade II distribution in a small aquatic continuum. Moving from relatively pristine sites toward areas with higher human disturbance, the abundance of clade II isolates and new *E. coli* genotypes reduces, while *E. coli* bearing class I integrons and belonging to CCs of public health concern accumulates. Altogether, this work exemplified that sampling of relatively pristine subtropical environments could lead to the discovery of more *E. coli* and cryptic clade diversity, which is imperative for understanding the extent of their genetic and functional variations in relation to diverse habitat conditions.

## Data Availability Statement

The datasets presented in this study can be found in online repositories. The names of the repository/repositories and accession number(s) can be found below: https://www.ncbi.nlm.nih.gov/genbank/, OM025257–OM030212.

## Author Contributions

XK: experiments, bioinformatics, analysis and interpretation, original draft, and review and editing. ZS: bioinformatics, analysis and interpretation, and original draft. CW, YY, HL, and SC: experiments, analysis and interpretation, and review and editing. JK: review and editing, and supervision. SL: review and editing, and supervision and funding acquisition. All authors contributed to the article and approved the submitted version.

## Conflict of Interest

The authors declare that the research was conducted in the absence of any commercial or financial relationships that could be construed as a potential conflict of interest.

## Publisher’s Note

All claims expressed in this article are solely those of the authors and do not necessarily represent those of their affiliated organizations, or those of the publisher, the editors and the reviewers. Any product that may be evaluated in this article, or claim that may be made by its manufacturer, is not guaranteed or endorsed by the publisher.

## Footnotes

1
http://enterobase.warwick.ac.uk
