# Supplementary material for: Genetic and Ecological Diversity of Escherichia coli and Cryptic Escherichia Clades in Subtropical Aquatic Environments
Source: Front Microbiol. 2022 Feb 17;13:811755. doi: 10.3389/fmicb.2022.811755 (PMC8891540; doi:10.3389/fmicb.2022.811755)
Supplement: Supplementary file 1 [file Table_1.docx]

**Supplementary file 1: Distribution of sampling location and site description for TK and KLH**

**Distribution of sampling location**

In general, the isolates used in this study were collected from 18 different locations, covering eastern and western Hong Kong, China (Figure S1). Isolates were mainly from the TK watershed located in western Hong Kong and the KLH intertidal mudflat in eastern Hong Kong.


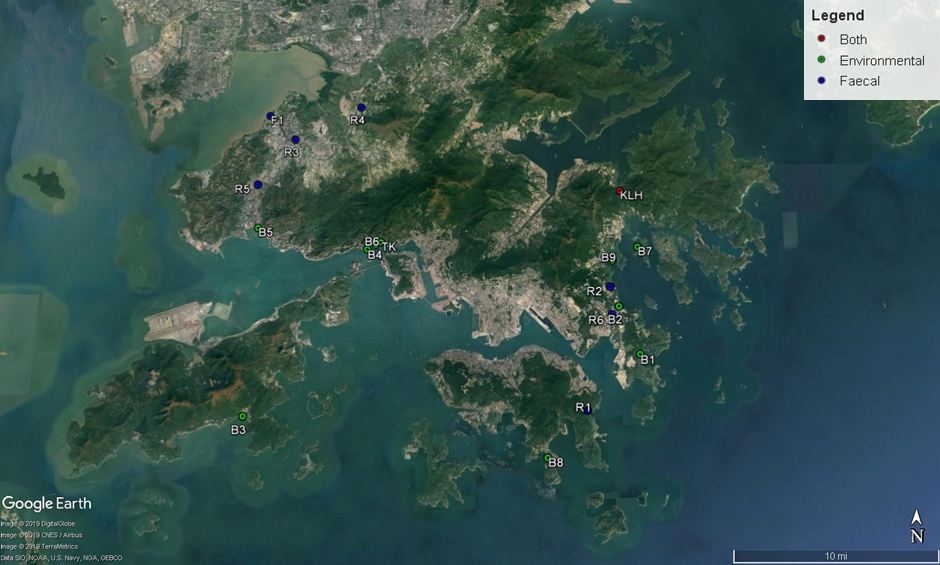


Figure S1. Distribution of sampling locations. R denotes residential area; B, beach; F, farm; TK, TK watershed; and KLH, KLH mudflat. Green legend denotes environmental samples were collected; blue, faecal; and red, both.

**TK watershed**

The TK watershed is a freshwater watershed connected to a beach in Tsuen Wan, Hong Kong, located to the west of Victoria Harbour. The sampled sites spanned a gradient of human disturbance from the relatively undisturbed streams and catchwater located upstream to the downstream village and beach area with relatively higher level of disturbance. The isolates used in this study were recovered from water, sediment, and periphyton samples from 13 sampling sites. *E. coli* was ubiquitous in the study area, with periphyton and sediment as environmental sinks of *E. coli* in the watershed. Upon perturbation (for example by storm event) these *E. coli* could be suspended into the overlaying water.

The 13 sampling sites were broadly divided into two categories (high and low impact) based on perceived human impact level observed on-site, such as distance from housing, beach activities, human and vehicle traffic (Figure S2). Six low human impact sites were situated upstream along the edge of a country park. Five of these sites were situated in three streams, with the remaining site (Site 12) a roadside drain receiving waters from the country park located uphill. Seven downhill sites were designated as high human impact sites. Site 4 was a catchwater of stormwater that was channelled from uphill, located in the vicinity of human settlement, including village houses and squatters, north of the TK village located downhill. Another three sites were stormwater outfalls (Storm outfall 1 and 2) and surface channel (Site 3) in the TK village, which is next to the TK beach. The two stormwater outfalls discharge directly into the beach. The remaining three sites were in the water column of the beach (Site 5, 6, and 7). The beach area is known to be affected by effluents of Stonecutters Island Sewage Treatment Work (SCISTW) as it is located just about 8 km to the northwest of the SCISTW effluent outfall. SCISTW is the largest sewage treatment plant in Hong Kong, commissioned as part of the Harbour Area Treatment Scheme initiated by the government of Hong Kong Special Administrative Region. Effluents treated by chemically enhanced primary treatment are discharged into the western part of Victoria Harbour (Chan *et al.*, 2013). The beach area was perceived to receive the highest level of human disturbance, including direct body contact (beach activities), influence by effluent from SCISTW, and possible leakage from failing sewage infrastructure or cross-connected sewage and stormwater networks.

Sample collection for dry season 2016 was done on 4^th^ and 11^th^ November 2016. Triplicate samples of water, sediment and periphyton were collected when present and accessible. Beach water samples were collected using sterile centrifuge tubes (50 ml each) mounted onto a telescopic rod. Water samples from other stations were collected using a similar method. Sediment and periphyton samples were collected using sterile spatulas or spades and transferred into sterile centrifuge tubes. All samples collected were kept on ice during transport to the lab. Samples were processed, filtered with membrane filtration technique and colonies were grown using ChromAgar^TM^ ECC (CHROMagar Microbiology, Paris, France) following the method of the Environmental Protection Department of Hong Kong (Ho and Tam, 1997).

A total of 258 isolates were selected for MLST, of which 103 and 155 isolates were from areas designated as low and high human impact, respectively. Only isolates from sediment and/or periphyton samples were tested for most sites. Isolates from all three matrices were analysed for storm outfall 1, which collects and discharges runoff from uphill, whereas only water samples were analysed for the beach samples. Six to ten isolates per sample matrix per site were tested. Not all sample matrices from each station were analysed for MLST (Table S1). We focused on sediments and periphyton as isolates from these matrices are more likely to be persistent in the environment instead of being transient. As the TK beach was a gazetted beach that the government routinely monitored for water quality, we focused on water column for the beach since water samples are analysed for beach water quality monitoring. As storm outfall 1 collected most water from the catchwater and streams in the watershed to be discharged into the beach, more isolates were selected for this site.

In a follow-up investigation following a typhoon event, additional water samples were collected from the beach and storm outfall 1 on 20^th^ and 21^st^ June 2017 (wet season). In total, 123 isolates were recovered, 39 from storm outfall 1 and 84 from the beach.


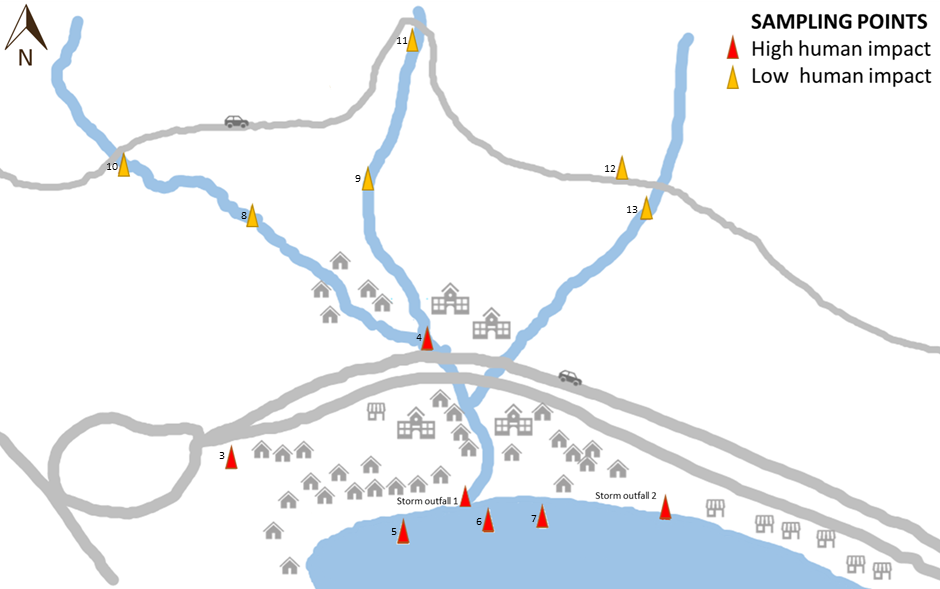


Figure S2. Sampling site depiction of the TK watershed. Figure not drawn to scale.

Table S1. Sample type, number of isolates analysed for MLST and prevalence of clade II-associated isolates for each sampling site in TK (dry season 2016).

| Human impact | Site | Sample type | No. of isolate | No. of clade II | % clade II |
| --- | --- | --- | --- | --- | --- |
| High | 1 | Water | 10 | 0 | 0.0 |
|  |  | Periphyton | 35 | 1 | 2.9 |
|  |  | Sediment | 34 | 1 | 2.9 |
|  |  | Total | 79 | 2 | 2.5 |
|  | 2 | Periphyton | 8 | 0 | 0.0 |
|  |  | Sediment | 9 | 0 | 0.0 |
|  |  | Total | 17 | 0 | 0.0 |
|  | 3 | Periphyton | 9 | 0 | 0.0 |
|  |  | Total | 9 | 0 | 0.0 |
|  | 4 | Periphyton | 10 | 0 | 0.0 |
|  |  | Sediment | 10 | 0 | 0.0 |
|  |  | Total | 20 | 0 | 0.0 |
|  | 5 | Water | 10 | 0 | 0.0 |
|  |  | Total | 10 | 0 | 0.0 |
|  | 6 | Water | 10 | 0 | 0.0 |
|  |  | Total | 10 | 0 | 0.0 |
|  | 7 | Water | 10 | 0 | 0.0 |
|  |  | Total | 10 | 0 | 0.0 |
|  |  | **TOTAL** | **155** | **2** | **1.3** |
| Low | 8 | Periphyton | 10 | 6 | 60.0 |
|  |  | Sediment | 10 | 0 | 0.0 |
|  |  | Total | 20 | 6 | 30.0 |
|  | 9 | Periphyton | 10 | 1 | 10.0 |
|  |  | Total | 10 | 1 | 10.0 |
|  | 10 | Periphyton | 10 | 1 | 10.0 |
|  |  | Sediment | 6 | 0 | 0.0 |
|  |  | Total | 16 | 1 | 6.3 |
|  | 11 | Periphyton | 10 | 1 | 10.0 |
|  |  | Sediment | 9 | 1 | 11.1 |
|  |  | Total | 19 | 2 | 10.5 |
|  | 12 | Periphyton | 10 | 4 | 40.0 |
|  |  | Sediment | 8 | 2 | 25.0 |
|  |  | Total | 18 | 6 | 33.3 |
|  | 13 | Periphyton | 10 | 0 | 0.0 |
|  |  | Sediment | 10 | 0 | 0.0 |
|  |  | Total | 20 | 0 | 0.0 |
|  |  | **TOTAL** | **103** | **16** | **15.5** |

**KLH intertidal mudflat**

The KLH mudflat was a triangle-shaped, semi-enclosed intertidal mudflat in Sai Kung, Hong Kong. The seaward margin of the mudflat faces north and is approximately 200 m wide. Its southeast margin (≈500 m) is bounded by a 320m high hill, and southwestern margin (≈500 m) bordered shrubland that is adjacent to a 250m high hill. Twenty-two houses were scattered around the shrubland, with the house closest to the mudflat being at least 100 m away. The mudflat is a sink for terrestrial runoff in the wet season due to the steep surrounding terrains. Observed faecal sources at the sampling site included a herd of approximately 15-20 feral cows and a few pet dogs. The maximum water depth of the mudflat is less than 2 m during high tides, whereas the sediment is fully exposed during low tides.

Samples were collected repeatedly from a single site in the mudflat over a period of eight months spanning May 2009 to October 2010. Sediment samples were collected during low tide while overlay seawater was collected during high tide, similar to the methods described for the TK sampling. Faecal samples were collected from faecal materials defecated by the feral cows. Isolates were prescreened by another genotyping method based on polymerase chain reaction (PCR), i.e. repetitive element fingerprinting using REP-PCR (repetitive element palindromic-PCR) primers (Mohapatra *et al.*, 2007) to obtain a smaller isolate collection that encompassed as much of the diversity of the original collection (>3000 isolates) as possible due to high cost of performing MLST on all isolates.


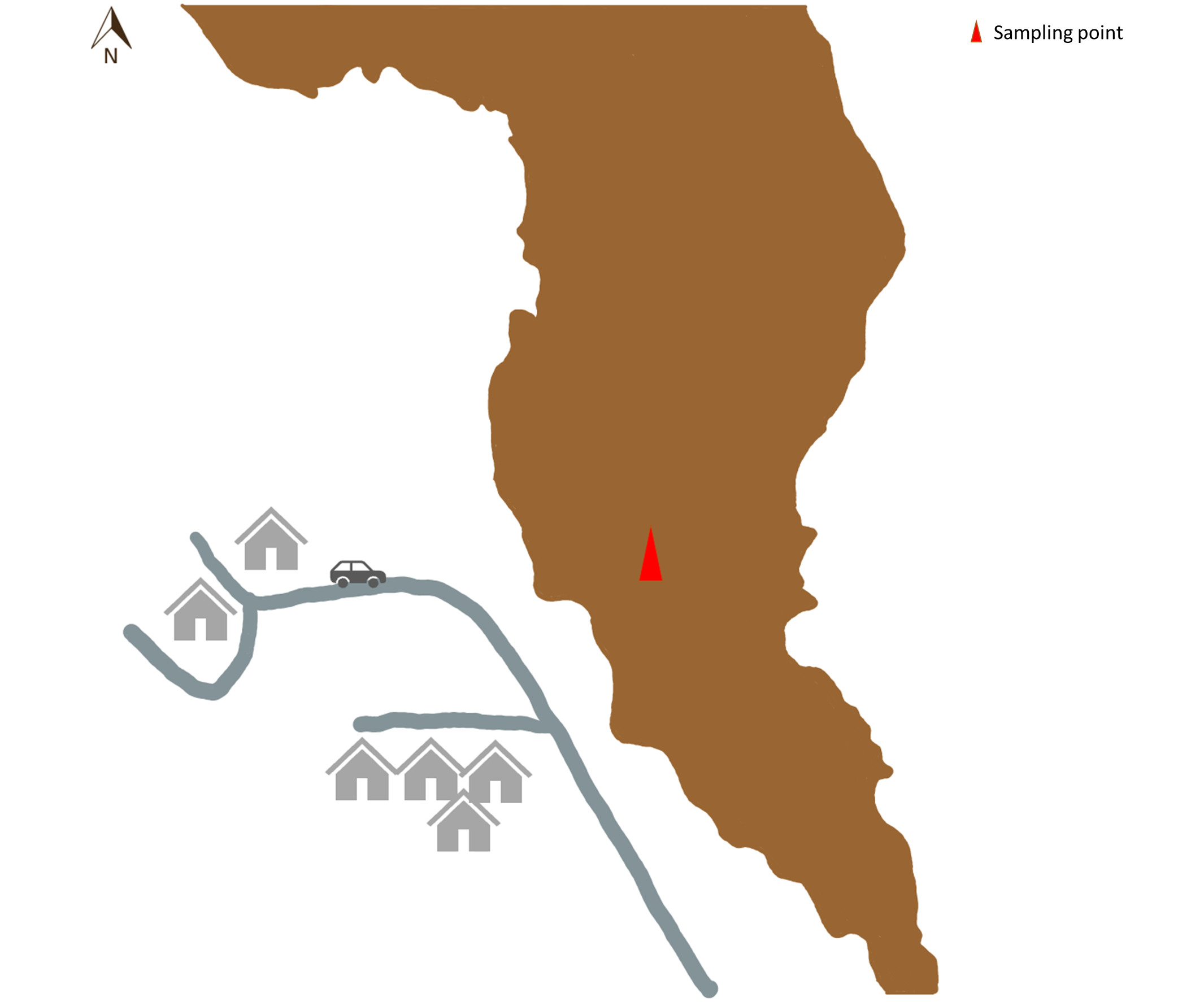


Figure S3. Sampling site depiction of the KLH mudflat. Figure not drawn to scale.

References:

Chan, S. N., Thoe, W., & Lee, J. H. (2013). Real-time forecasting of Hong Kong beach water quality by 3D deterministic model. *Water research*, *47*(4), 1631-1647.

Ho, B. S. W., & Tam, T. Y. (1997). Enumeration of *E*. *coli* in environmental waters and wastewater using a chromogenic medium. *Water science and technology*, *35*(11-12), 409-413.

Mohapatra, B. R., Broersma, K., & Mazumder, A. (2007). Comparison of five rep-PCR genomic fingerprinting methods for differentiation of fecal *Escherichia coli* from humans, poultry and wild birds. *FEMS microbiology letters*, *277*(1), 98-106.

**Supplementary file 2: Information of reference strains**

Table S2. Reference strains background information

| Strain | ST | Location | Sample Type | Phylogroup/ Cryptic Clade (I to VI)/ Species |
| --- | --- | --- | --- | --- |
| Ecor25 | 10 | U.S.A. | Dog | A |
| Ecor3 | 10 | U.S.A. | Dog | A |
| Ecor1 | 10 | U.S.A. | Human | A |
| Ecor11 | 10 | Sweden | Human | A |
| Ecor12 | 10 | Sweden | Human | A |
| Ecor14 | 10 | Sweden | Human | A |
| Ecor5 | 10 | U.S.A. | Human | A |
| Ecor8 | 10 | U.S.A. | Human | A |
| Ecor9 | 10 | Sweden | Human | A |
| Ecor10 | 43 | Sweden | Human | A |
| Ecor13 | 44 | Sweden | Human | A |
| Ecor15 | 45 | Sweden | Human | A |
| Ecor16 | 46 | U.S.A. | Feline | A |
| Ecor17 | 47 | Indonesia | Swine | A |
| Ecor20 | 48 | Bali | Bovine | A |
| Ecor21 | 48 | Bali | Bovine | A |
| Ecor18 | 48 | U.S.A. | Celebese ape | A |
| Ecor19 | 48 | U.S.A. | Celebese ape | A |
| Ecor2 | 49 | U.S.A. | Human | A |
| Ecor22 | 50 | Bali | Bovine | A |
| Ecor23 | 51 | U.S.A. | Elephant | A |
| Ecor24 | 52 | Sweden | Human | A |
| Ecor4 | 63 | U.S.A. | Human | A |
| Ecor43 | 65 | Sweden | Human | A |
| Ecor6 | 77 | U.S.A. | Human | A |
| Ecor7 | 87 | U.S.A. | Orangutan | A |
| Ecor26 | 53 | U.S.A. | Human | B1 |
| Ecor27 | 53 | U.S.A. | Giraffe | B1 |
| Ecor28 | 54 | U.S.A. | Human | B1 |
| Ecor29 | 55 | U.S.A. | Kangaroo rat | B1 |
| Ecor30 | 56 | Canada | Bison | B1 |
| Ecor32 | 56 | U.S.A. | Giraffe | B1 |
| Ecor33 | 56 | U.S.A. | Ovine | B1 |
| Ecor34 | 58 | U.S.A. | Dog | B1 |
| Ecor45 | 67 | Indonesia | Pig | B1 |
| Ecor58 | 75 | U.S.A. | Feline | B1 |
| Ecor67 | 84 | Indonesia | Goat | B1 |
| Ecor68 | 85 | U.S.A. | Giraffe | B1 |
| Ecor69 | 86 | U.S.A. | Celebese ape | B1 |
| Ecor71 | 88 | Sweden | Human | C |
| Ecor72 | 89 | Sweden | Human | C |
| Ecor53 | 12 | U.S.A. | Human | B2 |
| Ecor60 | 12 | Sweden | Human | B2 |
| Ecor51 | 73 | U.S.A. | Human | B2 |
| Ecor52 | 73 | U.S.A. | Orangutan | B2 |
| Ecor54 | 73 | U.S.A. | Human | B2 |
| Ecor56 | 73 | Sweden | Human | B2 |
| Ecor57 | 73 | U.S.A. | Gorilla | B2 |
| Ecor55 | 74 | Sweden | Human | B2 |
| Ecor59 | 76 | U.S.A. | Human | B2 |
| Ecor61 | 78 | Sweden | Human | B2 |
| Ecor62 | 79 | Sweden | Human | B2 |
| Ecor63 | 80 | Sweden | Human | B2 |
| Ecor64 | 81 | Sweden | Human | B2 |
| Ecor65 | 82 | U.S.A. | Celebese ape | B2 |
| Ecor66 | 83 | U.S.A. | Celebese ape | B2 |
| Ecor70 | 88 | U.S.A. | Gorilla | C |
| Ecor71 | 88 | Sweden | Human | C |
| Ecor72 | 89 | Sweden | Human | C |
| Ecor44 | 66 | U.S.A. | Cougar | D |
| Ecor46 | 68 | U.S.A. | Celebese ape | D |
| Ecor47 | 69 | Papua New Guinea | Sheep | D |
| Ecor48 | 70 | Sweden | Human | D |
| Ecor49 | 71 | Sweden | Human | D |
| Ecor50 | 72 | Sweden | Human | D |
| Ecor31 | 57 | U.S.A. | Feline | E |
| Ecor37 | 61 | U.S.A. | Marmoset | E |
| Ecor42 | 64 | U.S.A. | Human | E |
| Ecor35 | 59 | U.S.A. | Human | F |
| Ecor36 | 60 | U.S.A. | Human | F |
| Ecor38 | 62 | U.S.A. | Human | F |
| Ecor39 | 62 | Sweden | Human | F |
| Ecor40 | 62 | Sweden | Human | F |
| Ecor41 | 62 | Tonga | Human | F |
| H442 | 747 | Australia | Human | I |
| TW10509 | 747 | Guinea Bissau | Human | I |
| B827 | 2711 | Australia | Bird | I |
| M863 | 2715 | Australia | Mammal | I |
| E807 | 3692 | Australia | Environment | I |
| E1492 | 3720 | Australia | Environment | I |
| TW11930 | 5248 | Guinea Bissau | Human | I |
| ROAR019 | 5362 | Gabon | Yellow-backed duiker | II |
| B1147 | 5615 | Australia | Bird | II |
| EC5350 | 5615 | U.S.A. | Bird | II |
| B685 | 3568 | Australia | Bird | III |
| TW09276 | 3568 | U.S.A | Freshwater beach | III |
| TA004 | 3568 | Australia | Mammal | III |
| TW09231 | 5433 | U.S.A | Freshwater beach | III |
| TW09254 | 5433 | U.S.A | Freshwater beach | III |
| TW09266 | 5433 | U.S.A | Freshwater beach | III |
| B49 | 5358 | Australia | Bird | IV |
| TW14182 | 7531 | U.S.A | Freshwater beach | IV |
| TW11588 | 7533 | Puerto Rico | Soil | IV |
| Z205 | 125 | Germany | Parrot | V |
| RL325/96 | 133 | Germany | Dog | V |
| E1118 | 2721 | Australia | Environment | V |
| E1195 | 5260 | Australia | Environment | V |
| TW14264 | 5284 | U.S.A. | Surface water | V |
| E471 | 5443 | Australia | Environment | V |
| M1108 | 5500 | Australia | Mammal | V |
| TW14263 | 5566 | U.S.A. | Racoon | V |
| B1225 | 5600 | Australia | Bird | V |
| TW09308 | 6500 | U.S.A. | Freshwater beach | V |
| EC7003 | 6564 | U.S.A. | Dog | VI |
| *E. albertii* KF1 | 4638 | Poland | Human | *E. albertii* |
| *E. fergusonii* ATCC 35469 | 5298 | U.S.A. | Human | *E. fergusonii* |
| *S. enterica* Typhimurium LT2 |  | United Kingdom | Human | *Salmonella enterica* |

References:

Clermont, O., Gordon, D., & Denamur, E. (2015). Guide to the various phylogenetic classification schemes for *Escherichia coli* and the correspondence among schemes. *Microbiology*, *161*(5), 980-988.

Gangiredla, J., Mammel, M. K., Barnaba, T. J., Tartera, C., Gebru, S. T., Patel, I. R., ... & Lacher, D. W. (2018). Draft Genome Sequences of *Escherichia albertii*, *Escherichia fergusonii*, and Strains Belonging to Six Cryptic Lineages of *Escherichia* spp. *Genome Announc.*, *6*(18), e00271-18.

McClelland, M., Sanderson, K. E., Spieth, J., Clifton, S. W., Latreille, P., Courtney, L., ... & Hou, S. (2001). Complete genome sequence of *Salmonella enterica* serovar Typhimurium LT2. *Nature*, *413*(6858), 852.

Ochman, H., & Selander, R. K. (1984). Standard reference strains of *Escherichia coli* from natural populations. *Journal of bacteriology*, *157*(2), 690-693.

Walk, S. T., Alm, E. W., Gordon, D. M., Ram, J. L., Toranzos, G. A., Tiedje, J. M., & Whittam, T. S. (2009). Cryptic lineages of the genus *Escherichia*. *Applied and environmental microbiology*, *75*(20), 6534-6544.

Waters, N. R., Abram, F., Brennan, F., Holmes, A., & Pritchard, L. (2020). Easy phylotyping of *Escherichia coli* via the EzClermont web app and command-line tool. *Access microbiology*, *2*(9).

Wirth, T., Falush, D., Lan, R., Colles, F., Mensa, P., Wieler, L. H., ... & Achtman, M. (2006). Sex and virulence in *Escherichia coli*: an evolutionary perspective. *Molecular microbiology*, *60*(5), 1136-1151.

**Supplementary file 3: Habitat spectra and geographical origins of the genotypes for each ecologically distinct group inferred by AdaptML analysis**


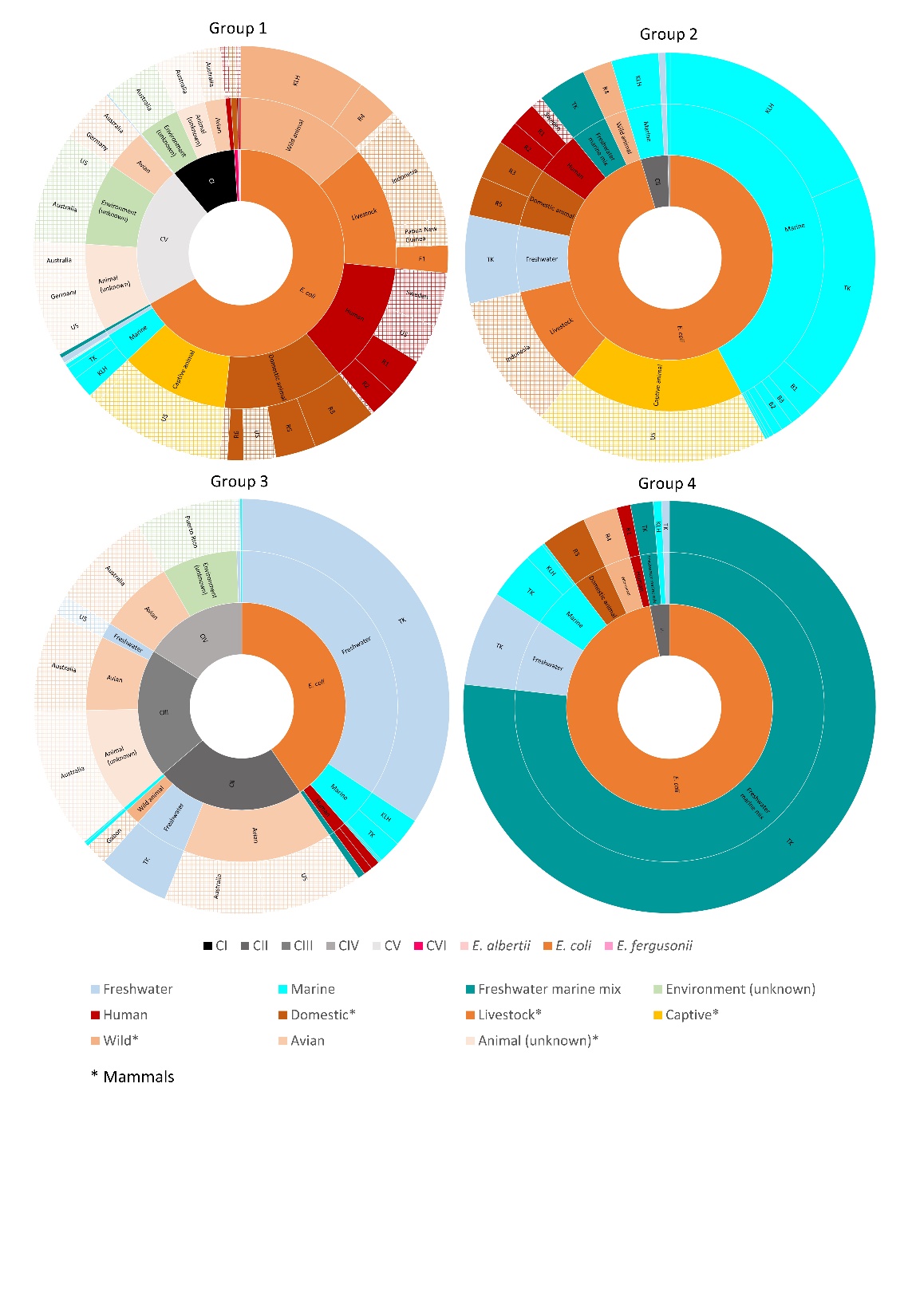


Figure S4. Habitat spectra and geographical origins of the genotypes for each ecologically distinct group inferred by AdaptML. Segments containing entries from other studies were filled with grid patterns.

**Supplementary file 4: Genetic relatedness among sequence types (STs)**

**
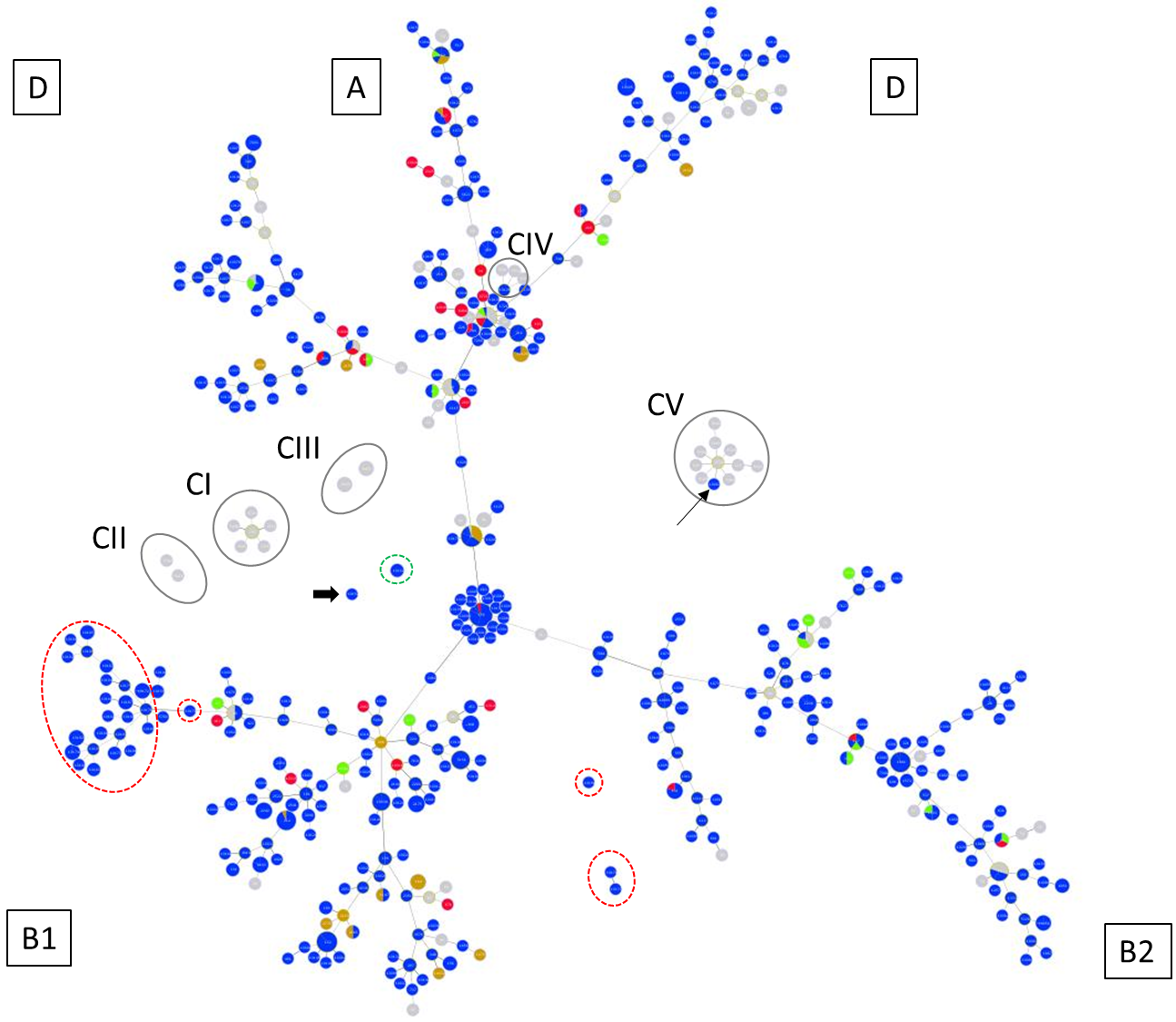
**

Figure S5. Minimum spanning tree (MST) generated with goeBURST algorithm as implemented in Phyloviz (Nascimento *et al.*, 2016). STs were linked only when there is at least an allele in common at one locus. Each node represented one ST. Node size is proportionate to the number of isolates contained in the node. Linkage distance (number of loci difference) not according to scale. Reference STs in Table S2 (except EC5350 and EC7003) were included. The cryptic clades references were indicated (CI to CIV). Blue: environmental isolates; red: human, faecal; brown: feral cow, faecal; green: dog, faecal; and grey: reference). Phylogroup for majority of the STs in each main cluster was indicated. Nodes encapsulated in red dashed line were the STs of cryptic clade II-associated isolates in this study and shared no common allele with the two clade II reference STs. One clade IV ST shared a common allele with clade IV reference STs at *mdh* (allele 56) and connected to the tree at *recA* (allele 2). Another clade IV ST shared no common allele with neither references nor other isolates thus detached from the tree (single node encapsulated in green dash line). The sole clade V in this study (thin arrow) is a double locus variant of ST2721, one of the reference clade V ST. One *E. coli* ST (thick arrow) shared no common allele with any other STs.


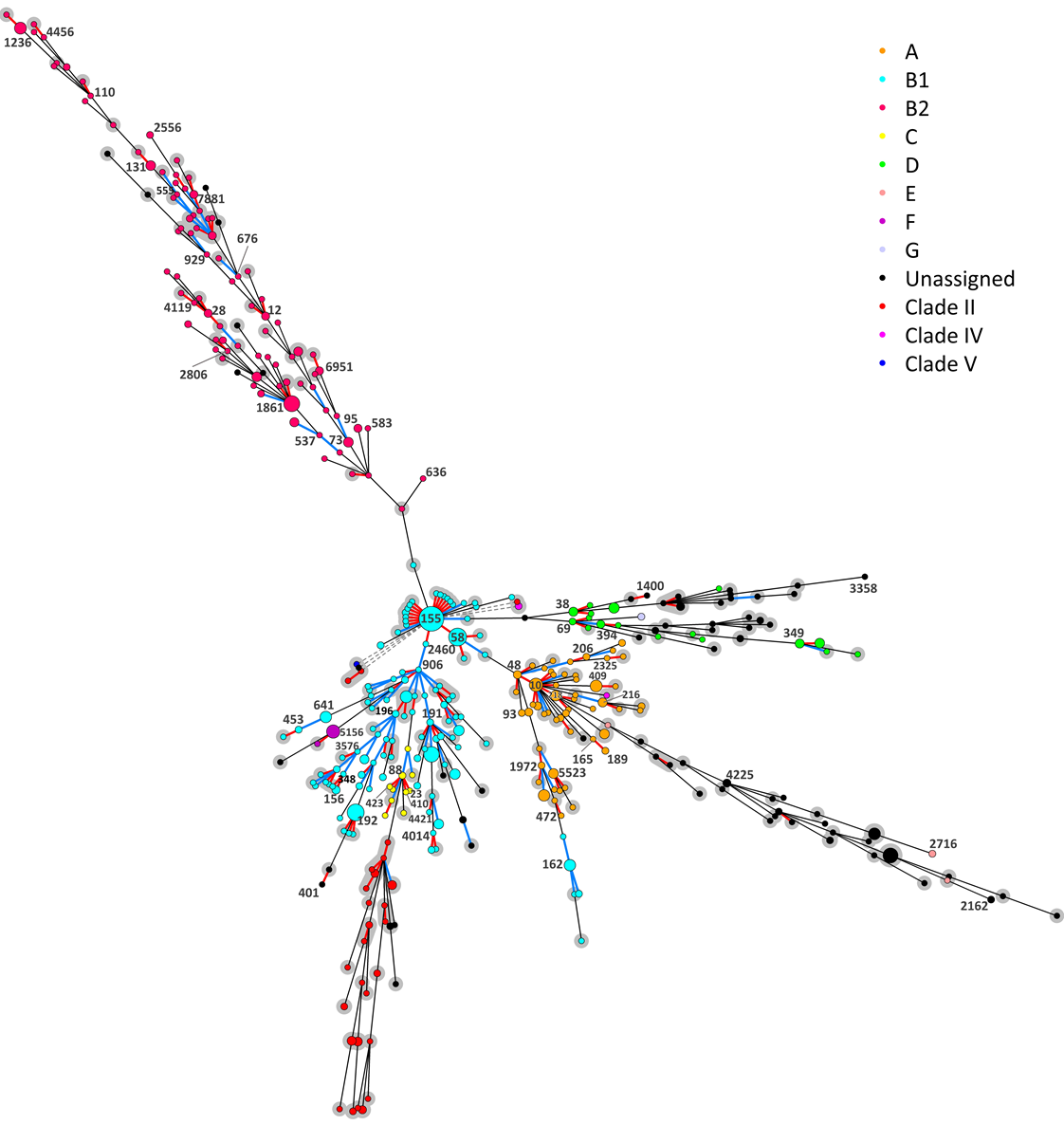


Figure S6. Minimum spanning tree (MST) displaying the genetic relatedness of the 708 isolates and overlaid with phylogroup information. Node size is proportionate to the number of isolates contained in the node. Branch length is proportionate to distance between nodes, with red branch denoted 1 locus difference; blue: 2 loci; black: 3 – 6 loci; and grey dashed branch: no loci in common. Novel STs were shaded with grey background and several known *E. coli* STs that could be identified in this study were indicated.

**Supplementary file 5: Genetic differentiation assessment using F_ST_**


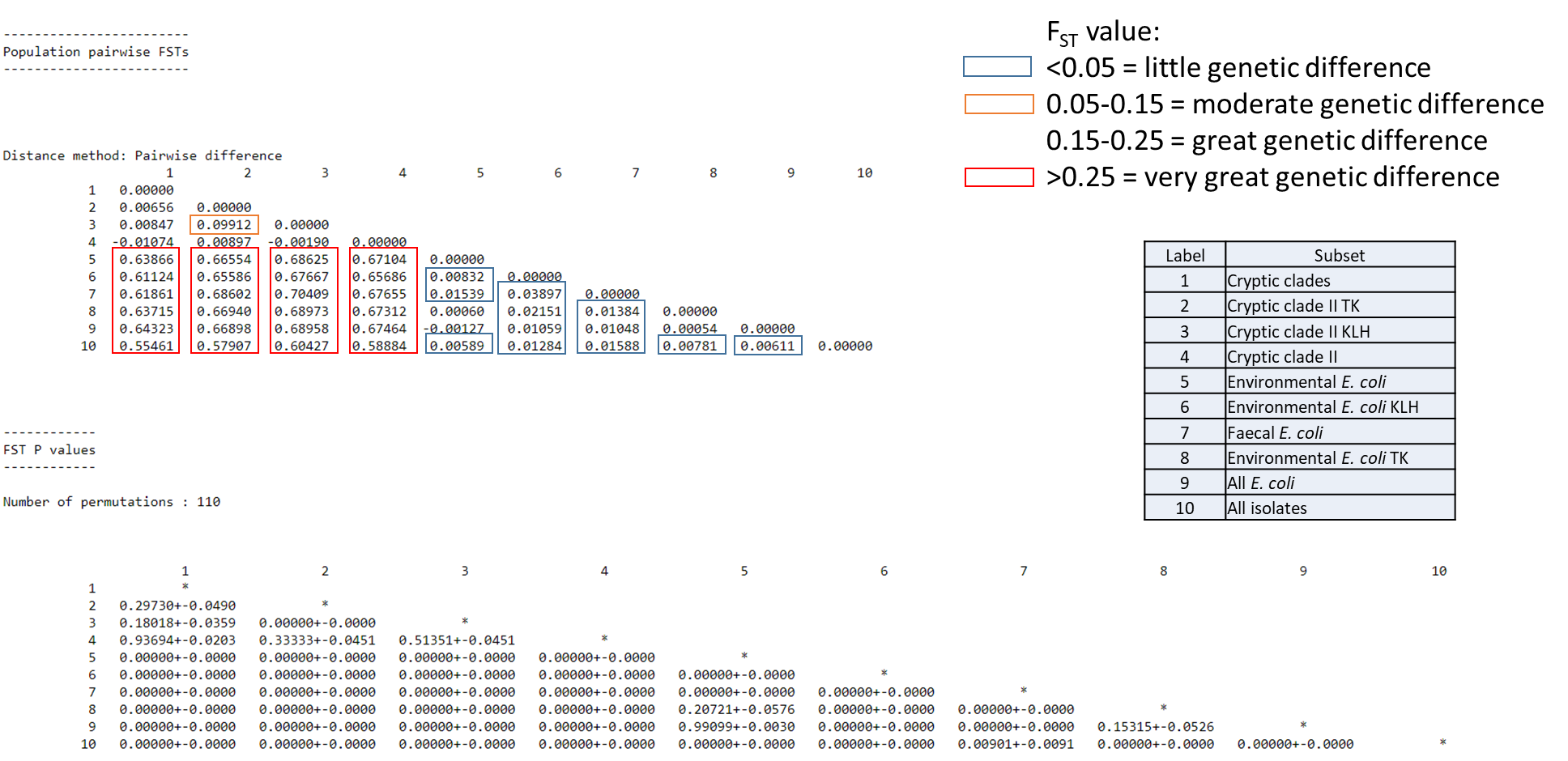


**Supplementary file 6: Relative abundance of cryptic clades of *Escherichia* in different studies**

Table S3. Comparison of the relative abundance of cryptic *Escherichia* clades in various aquatic environments

| Location | | Sample | No. of isolates | Cryptic clades abundance (%) | | | | | | Reference |
| --- | --- | --- | --- | --- | --- | --- | --- | --- | --- | --- |
|  |  |  |  | over total isolates | among all cryptic clades | | | | |  |
|  |  |  |  |  | CI | CII | CIII | CIV | CV |  |
| Freshwater beaches, Michigan, USA | | Water and sediment | 196 | 3.1 | 0 | 0 | 66.7 | 16.7 | 16.7 | Walk *et al*., 2007 |
| Hinkson Creek, Missouri, USA | | Water | 190 | 0.5 | 0 | 0 | 0 | 0 | 1 | Deng *et al*., 2014 |
| Adriatic Sea, Italy | | Coastal marine sediment | 138 | 14.5 | 0 | 0 | 5.0 | 5.0 | 90.0 | Vignaroli *et al*., 2015* |
| Lagoon of Venice, Italy | | Macroalgae and water | 287 | 2.7 | 87.5^#^ | | 0 | 0 | 12.5 | Quero *et al*., 2015 |
| Aquatic continuum composed of four rivers, France | Selles | Water | 99 | 5.1 | reported collectively as CII, CIII and CV | | | | | Petit *et al.*, 2017 |
|  |  | Sediment | 49 | 10.2 |  |  |  |  |  |  |
|  |  | All | 148 | 6.8 |  |  |  |  |  |  |
|  | Sébec | Water | 99 | 22.2 |  |  |  |  |  |  |
|  |  | Sediment | 59 | 40.0 |  |  |  |  |  |  |
|  |  | All | 158 | 28.5 |  |  |  |  |  |  |
|  | Tourville | Water | 96 | 9.4 |  |  |  |  |  |  |
|  |  | Sediment | 59 | 16.9 |  |  |  |  |  |  |
|  |  | All | 155 | 12.3 |  |  |  |  |  |  |
|  | Risle | Water | 94 | 1.1 |  |  |  |  |  |  |
|  |  | Sediment | 96 | 5.2 |  |  |  |  |  |  |
|  |  | All | 190 | 3.2 |  |  |  |  |  |  |
| Tsuen Wan, Hong Kong | | Water | 40 | 0 | 0 | 0 | 0 | 0 | 0 | This study^$^ |
|  |  | Periphyton | 122 | 11.5 | 0 | 100 | 0 | 0 | 0 |  |
|  |  | Sediment | 96 | 4.2 | 0 | 100 | 0 | 0 | 0 |  |
|  |  | All | 258 | 7 | 0 | 100 | 0 | 0 | 0 |  |

* the isolates were originally from two separate studies done by Luna *et al.* (2010) and Vignaroli *et al.* (2013)

# results in the article were expressed as clade I or II

$ isolates from the dry season 2016 sampling campaign

Deng, D., Zhang, N., Mustapha, A., Xu, D., Wuliji, T., Farley, M., ... & Zheng, G. (2014). Differentiating enteric *Escherichia* *coli* from environmental bacteria through the putative glucosyltransferase gene (ycjM). *Water research*, *61*, 224-231.

Luna, G. M., Vignaroli, C., Rinaldi, C., Pusceddu, A., Nicoletti, L., Gabellini, M., ... & Biavasco, F. (2010). Extraintestinal *Escherichia* *coli* carrying virulence genes in coastal marine sediments. *Applied and environmental microbiology*, *76*(17), 5659-5668.

Petit, F., Clermont, O., Delannoy, S., Servais, P., Gourmelon, M., Fach, P., ... & Berthe, T. (2017). Change in the structure of *Escherichia* *coli* population and the pattern of virulence genes along a rural aquatic continuum. *Frontiers in microbiology*, *8*, 609.

Quero, G. M., Fasolato, L., Vignaroli, C., & Luna, G. M. (2015). Understanding the association of *Escherichia* *coli* with diverse macroalgae in the lagoon of Venice. *Scientific reports*, *5*, 10969.

Vignaroli, C., Di Sante, L., Magi, G., Luna, G. M., Di Cesare, A., Pasquaroli, S., ... & Biavasco, F. (2015). Adhesion of marine cryptic *Escherichia* isolates to human intestinal epithelial cells. *The ISME journal*, *9*(2), 508.

Vignaroli, C., Luna, G. M., Pasquaroli, S., Di Cesare, A., Petruzzella, R., Paroncini, P., & Biavasco, F. (2013). Epidemic *Escherichia* *coli* ST131 and *Enterococcus* *faecium* ST17 in coastal marine sediments from an Italian beach. *Environmental science & technology*, *47*(23), 13772-13780.

Walk, S. T., Alm, E. W., Calhoun, L. M., Mladonicky, J. M., & Whittam, T. S. (2007). Genetic diversity and population structure of *Escherichia* *coli* isolated from freshwater beaches. *Environmental microbiology*, *9*(9), 2274-2288.
